# Reciprocal crosstalk between Th17 and mesothelial cells promotes metastasis‐associated adhesion of ovarian cancer cells

**DOI:** 10.1002/ctm2.1604

**Published:** 2024-04-02

**Authors:** Felix Neuhaus, Sonja Lieber, Veronika Shinkevich, Anna Mary Steitz, Hartmann Raifer, Kathrin Roth, Florian Finkernagel, Thomas Worzfeld, Andreas Burchert, Corinna Keber, Andrea Nist, Thorsten Stiewe, Silke Reinartz, Vanessa M. Beutgen, Johannes Graumann, Kim Pauck, Holger Garn, Matthias Gaida, Rolf Müller, Magdalena Huber

**Affiliations:** ^1^ Institute of Systems Immunology Center for Tumor Biology and Immunology (ZTI) Philipps University Marburg Germany; ^2^ Department of Translational Oncology Center for Tumor Biology and Immunology (ZTI) Philipps University Marburg Germany; ^3^ Institute of Pharmacology Philipps University Marburg Germany; ^4^ FACS Core Facility Center for Tumor Biology and Immunology (ZTI) Philipps University Marburg Germany; ^5^ Cell Imaging Core Facility, Center for Tumor Biology and Immunology (ZTI) Philipps University Marburg Germany; ^6^ Bioinformatics Core Facility, Center for Tumor Biology and Immunology (ZTI) Philipps University Marburg Germany; ^7^ Department of Pharmacology Max Planck Institute for Heart and Lung Research Bad Nauheim Germany; ^8^ Department of Hematology Oncology and Immunology University Hospital Giessen and Marburg Marburg Germany; ^9^ Comprehensive Biomaterial Bank Marburg (CBBMR) and Institute of Pathology Philipps University Marburg Germany; ^10^ Genomics Core Facility Institute of Molecular Oncology Member of the German Center for Lung Research (DZL) Philipps University Marburg Germany; ^11^ Institute of Translational Proteomics and Translational Proteomics Core Facility Biochemical Pharmacological Centre Philipps University Marburg Germany; ^12^ Translational Inflammation Research Division and Core Facility for Single Cell Multiomics Philipps University Marburg Germany; ^13^ Institute of Pathology University Medical Center Mainz, Johannes Gutenberg University Mainz Germany; ^14^ TRON, Translational Oncology at the University Medical Center Johannes Gutenberg University Mainz Germany; ^15^ Research Center for Immunotherapy University Medical Center Mainz, Johannes Gutenberg University Mainz Germany

**Keywords:** mesothelial cells, metastasis, ovarian carcinoma, Th17 cells

## Abstract

**Background:**

IL‐17A and TNF synergistically promote inflammation and tumorigenesis. Their interplay and impact on ovarian carcinoma (OC) progression are, however, poorly understood. We addressed this question focusing on mesothelial cells, whose interaction with tumor cells is known to play a pivotal role in transcoelomic metastasis formation.

**Methods:**

Flow‐cytometry and immunohistochemistry experiments were employed to identify cellular sources of IL‐17A and TNF. Changes in transcriptomes and secretomes were determined by bulk and single cell RNA sequencing as well as affinity proteomics. Functional consequences were investigated by microscopic analyses and tumor cell adhesion assays. Potential clinical implications were assessed by immunohistochemistry and survival analyses.

**Results:**

We identified Th17 cells as the main population of IL‐17A‐ and TNF producers in ascites and detected their accumulation in early omental metastases. Both IL‐17A and its receptor subunit IL‐17RC were associated with short survival of OC patients, pointing to a role in clinical progression. IL‐17A and TNF synergistically induced the reprogramming of mesothelial cells towards a pro‐inflammatory mesenchymal phenotype, concomitantly with a loss of tight junctions and an impairment of mesothelial monolayer integrity, thereby promoting cancer cell adhesion. IL‐17A and TNF synergistically induced the Th17‐promoting cytokines IL‐6 and IL‐1β as well as the Th17‐attracting chemokine CCL20 in mesothelial cells, indicating a reciprocal crosstalk that potentiates the tumor‐promoting role of Th17 cells in OC.

**Conclusions:**

Our findings reveal a novel function for Th17 cells in the OC microenvironment, which entails the IL‐17A/TNF‐mediated induction of mesothelial‐mesenchymal transition, disruption of mesothelial layer integrity and consequently promotion of OC cell adhesion. These effects are potentiated by a positive feedback loop between mesothelial and Th17 cells. Together with the observed clinical associations and accumulation of Th17 cells in omental micrometastases, our observations point to a potential role in early metastases formation and thus to new therapeutic options.

## Graphical abstract

A decisive step in ovarian carcinoma metastasis is breaching the protective mesothelial monolayer covering the peritoneal organs. This study has uncovered a hitherto unknown mechanism involving the induction of mesenchymal reprogramming by the synergistic action of IL‐17A and TNF produced by Th17 cells, which renders the mesothelial monolayer susceptible to cancer cell adhesion. These reprogrammed mesothelial cells also secrete Th17‐promoting mediators, thereby establishing a positive feedback loop that amplifies the impact of IL‐17A and TNF on mesothelial cells. Disruption of this reciprocal crosstalk may represent a promising strategy to interfere with metastatic spreading.

Ovarian carcinoma metastasis is promoted by reciprocal crosstalk of Th17, mesothelial and cancer cells.

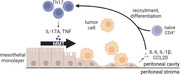

## 1 BACKGOUND

Ovarian cancer (OC) is the fifth leading cause of cancer‐related death in women, with an estimated 13,270 deaths in the US in 2023.^1^ High‐grade serous ovarian cancer represents the most common and most aggressive subtype with an overall 5‐year survival rate of approximately 35% due to a diagnosis at advanced stages, its recurrence after first‐line therapy and the development of chemoresistance.^2^ HGSC spreads primarily via the peritoneal fluid, which typically increases to large volumes during disease progression (ascites), further enhancing passive transcoelomic cancer cell dissemination.^3^ Ascites contains abundant detached tumor cells, tumor‐cell spheroids and various host cell types, including innate and adaptive immune cells, thereby constituting a crucial component of the tumor microenvironment (TME) besides the solid tumor foci. All cell types of the TME secrete factors into the ascites, reciprocally modulating their activation, differentiation and function, thereby establishing loops that promote tumor progression and suppress anti‐tumor immune surveillance.^4^

Transcoelomic tumor cell seeding requires attachment to, and breaching of the peritoneum lining the peritoneal cavity and abdominal organs. This includes the omentum, which is the preferred site of OC metastases, presumably by providing a rich source of fatty acid nutrients and tumor‐promoting factors secreted by adipocytes, fibroblasts and immune cells.^4^, ^5^, ^6^, ^7^, ^8^ The peritoneum is composed of a monolayer of flat mesothelial cells on a thin layer of connective tissue. The mesothelium faces the peritoneal cavity and produces a lubricating fluid that provides a slippery, non‐adhesive surface to facilitate intracoelomic movement. Due to these properties the mesothelium poses a physical barrier to tumor cells.^9^ A crucial step in the formation of tanscoelomic metastases is, therefore, the disruption of the mesothelial monolayer to allow tumor cells to gain access to the submesothelial environment.^9^

Tumor cells modify the mesothelial monolayer through direct contact and via secreted soluble factors. OC spheroids, for example, caused clearance of mesothelial cells by myosin‐dependent traction force.^10^ Another proposed mechanism is the induction of a mesenchymal phenotype in mesothelial cells in response to TGF‐β1 secretion by tumor cells. This mesothelial‐mesenchymal transition (MMT) included augmented fibronectin production, which in turn promoted tumor cell adhesion, invasion, and proliferation.^11^ Furthermore, apoptosis of mesothelial cells induced by tumor cells^12^ or tumor‐associated host cells^13^ has been proposed as a mechanism of peritoneal invasion. Collectively, the mechanisms uncovered in these studies reveal a reciprocal crosstalk between tumor and mesothelial cells in metastasis formation.

The function of mesothelial cells, their monolayer integrity and their crosstalk with tumor cells are further influenced by ascites. It was, for instance, found to induce the production of angiopoietin‐like 4 (ANGPTL4), which promotes MMT and early metastases formation.^14^ Furthermore, inflammatory cytokines present in ascites, including TNF, IL‐1β and IL‐6, have been demonstrated to reprogram mesothelial cells towards a mesenchymal phenotype, which induces retraction of the protective mesothelial cells, thereby exposing the underlying collagen‐rich matrix to attaching tumor cells.^9^, ^15^ Elevated levels of TNF in OC ascites have also been associated with elevated expression of the adhesion molecule VCAM‐1 on mesothelial cells, thereby enabling cancer‐mesothelial cell interactions leading to cancer cell attachment and invasion.^16^ Additionally, TNF produced by OC cells enhances inflammation by stimulating production of cytokines, chemokines and angiogenic factors to promote tumor cell dissemination.^17^ In line with these observations, the tumor‐promoting lipid mediator lysophosphatidic acid induces TNF to regulate a pro‐inflammatory cytokine network in OC.^18^ TNF has also been reported to upregulate IL‐17A production by CD4^+^ T cells, thereby recruiting myeloid cells into the TME and enhancing tumor growth. Consistent with this finding, neutralization of TNF by infliximab reduced IL‐17A plasma levels in patients with advanced OC, indicating a pro‐inflammatory TNF‐IL‐17A axis.^19^

Similar to the action of TNF,^20^ increased levels of IL‐17A drive chronic inflammation during, for example, autoimmune reactions of the skin, joints, or the central nervous system.^21^ IL‐17A homodimers and IL‐17A/F heterodimers signal through the receptor IL‐17R consisting of two chains, the specific IL‐17RC and the common chain IL‐17RA.^21^ IL‐17A is a potent proinflammatory cytokine that promotes tumor progression through both direct and indirect mechanisms,^22^ and its impact has been studied in a plethora of cancer entities, including lung, pancreatic and ovarian carcinoma cells^19^, ^23^, ^24^, ^25^, ^26^ In OC, the role of IL‐17A remains controversial as potential anti‐tumor functions have also been reported.^27^, ^28^

Different cell types produce IL‐17A, CD4^+^, CD8^+^, γδT cells and various innate immune cell populations, including mucosal‐associated invariant T (MAIT) cells, termed Th17, Tc17, γδΤ17 and MAIT17 cells, respectively. All of these cells are characterized by the expression of the transcription factor RORγt, production of IL‐17A/F and other pro‐inflammatory cytokines, e.g., IFNγ and TNF.^21^ In the context of OC, the role of IL‐17A producers seems to be complex and dependent on the compartment, species, and tumor stage.^27^, ^29^, ^30^, ^31^

IL‐17A has also been reported to act synergistically with TNF in inflammation,^32^, ^33^ recruitment of myeloid cells,^19^, ^34^ autoimmunity^35^, ^36^ and tumorigenesis.^23^ However, potential cooperative effects of IL‐17A and TNF in the regulation of mesothelial cell functions or OC progression remain, however, to be addressed. In the present study, we discovered an IL‐17A/TNF‐driven amplification loop involving mesothelial and Th17 cells, which promoted OC cell adhesion to the mesothelial monolayer, suggesting an involvement in early omental metastases formation.

## 2 METHODS AND MATERIALS

### 2.1 Biomaterials from OC patients

Ascites, peripheral blood mononuclear cells (PBMCs) and greater omentum tissue with metastatic lesions were collected from individuals diagnosed with high‐grade serous ovarian cancer (HGSC) at Marburg University Hospital. The acquisition and examination of human specimens received ethical clearance from the ethics committee at Philipps University (reference number 205/10). Donors provided written consent in accordance with the Declaration of Helsinki. The clinical data for all patients and the use of patient‐derived biomaterials are listed in Table S1.

Peritoneal mesothelial cells were obtained from the omentum involved a 30‐minute digestion of macroscopically tumor‐free omental tissue using trypsin. The resulting suspension containing all cell types except adipocytes was subjected to magnetic‐activated cell sorting (MACS) to remove CD45^+^ and EpCAM^+^ cells by negative selection. These cells were directly cryopreserved for bulk RNA‐Seq or cultured in OCMI/5% FCS medium for a maximum of three to five passages, as previously described.^8^ These cultured cells consisted of >95% mesothelial cells.^8^, ^13^ Tumor cell cultures were established from ascites tumor spheroids as described.^37^, ^38^

Tumor‐associated lymphocytes (TALs) from ascites were separated from other cell types, including tumor‐associated macrophages (TAMs) and tumor cells according to the published protocol.^8^ Briefly, mononuclear cells were separated by Lymphocyte Separation Medium 1077 (PromoCell, Heidelberg, Germany) and density gradient centrifugation. Subsequently, tumor spheroids were removed using 30 μ m and 100 μ m cell strainers. Finally, CD14^+^ TAMs were removed by magnetic activated cell sorting (MACS) according to the supplier's standard protocols (Miltenyi Biotec, Bergisch Gladbach, Germany). The TAL fraction obtained by this procedure contained less than 3.5% EpCAM^+^ cells.

PBMCs were obtained from patients by density gradient centrifugation using Pancoll (PAN Biotech, Aidenbach, Germany).

### 2.2 FACS analysis of IL‐17/TNF‐producing cells

CD14^−^ ascites cells or PBMCs were cultured for 16 h in RPMI/ 10% AB serum and restimulated with ionomycin (1 ng/ml), PMA (100 nmol/ml) and α ‐CD28 (1 ng/ml). After 2 h incubation, 5 μ g/ml brefeldin A was added. At the experimental endpoint, cells were measured after immunostaining for life/dead, CD4, CD8, TCRV α 7.2, TCRγδ, CD161, IFNγ, IL‐17A and TNF (FACS Aria III; BD Biosciences, Franklin Lakes, USA). Cells were analyzed by flow cytometry (FACS Diva) and FlowJo V10.1 software. The following antibodies were used: α‐CD4 APC (300552, Biolegend, San Diego, USA), α‐CD8 BV510 (344732, Biolegend, San Diego, USA), α‐TCRVα7.2 PerCP Cy5.5 (351710, Biolegend, San Diego, USA), α‐TCRγδ PECy7 (655410, BD Bioscience), α‐CD161 BV421 (339914, Biolegend, San Diego, USA), α‐IFNγ AF488 (502515, Biolegend, San Diego, USA), α‐IL17A PE (512306, Biolegend, San Diego, USA), α‐TNF Dazzle (502946, Biolegend, San Diego, USA), Zombie NIR (423105, Biolegend, San Diego, USA).

### 2.3 Immunohistochemistry

Samples of omental tissue with micrometastasis from OC patients were provided by the Department of Pathology (Marburg University Hospital). After deparaffinization, rehydration and antigen retrieval at pH 6, the following antibodies were used for double staining: anti‐hCD4 (Agilent, IR649), anti‐hRORγt (LS‐B4659‐100, Biozol, Eiching, Germany), and anti‐hIL‐17A (MAB3171, Biotechne, Minneapolis, USA). DAKO Envision Flex system (brown, Agilent, Santa Clara, USA) and permanent Green (Zytomed, Baden‐Bade, Germany) were used for visualization. CD4+IL‐17A^+^, CD4^+^RORγt^+^, and total CD4^+^ cells were counted per mm^2^. Images were taken with a Gryphax Subra camera (Jenoptik, Jena, Germany). Quantification was done using the ImageJ software.

### 2.4 Culture and stimulation of omentum‐derived mesothelial cells

For RNA analyses and affinity proteomics of conditioned medium (CM) mesothelial cells (see Biomaterials above) were seeded in OCMI/5% FCS medium. After 24 h the medium was changed and 30 ng/ml of rhIL‐17A (200‐17, Peprotech, Cranbury, USA), 1 ng/ml of rhTNF (300‐01A, Peprotech, Cranbury, USA) or 30 of ng/ml rhIL17A + 1 ng/ml rhTNF were added (rh: recombinant human). CM wand cells were harvested after 48 h of treatment. For treatment of CD4^+^ T cells with CM, mesothelial cells were seeded in OCMI medium with or without stimulus (30 ng/ml rhIL17A + 1 ng/ml rhTNF) for 48 h. Thereafter, the cells were washed and the medium was changed to RPMI/ 5% FCS with or without stimulus (30 ng/ml IL‐17A + 1 ng/ml TNF). CM was collected 24 h later. The conditions for cytokine treatment are based on previous publications,^17^, ^26^, ^39^ titrations and kinetic experiments.

### 2.5 cDNA synthesis and RT‐qPCR analysis

RNA isolation from cultured mesothelial cells was carried out using the NucleoSpin RNA kit (Macherey‐Nagel, Düren, Germany). For cDNA synthesis, the RevertAid first strand cDNA synthesis kit (Thermo Fisher Scientific, Waltham, MA, USA) was used according to the manufacturer's protocol. RT‐qPCR analysis of MMT marker genes was performed using iTaq Universal SYBR Green (BioRad, Hercules, USA) on a StepOnePlus instrument (Thermo Fisher Scientific, Waltham, USA). The qPCR reaction setup included optimized primers for standardization (Table S2).

### 2.6 Immunofluorescence analysis of mesothelial cells

Mesothelial cells (1.8×10^4^/well) were seeded in µ‐Slides (Ibidi, Gräfelfing, Germany) and stimulated for four days (30 ng/ml rhIL‐17A, 1 ng/ml rhTNF, 30 ng/ml rhIL17A + 1 ng/ml rhTNF). After two days, medium and cytokines were renewed. At the experimental endpoint, the monolayer was stained with anti‐ZO‐1 (339188, Invitrogen, Waltham, USA), phalloidin (23103, AAT Bioquest, Pleasanton, USA) and Hoechst (17535, AAT Bioquest, Pleasanton, USA). Images were taken on a Leica SP8i microscope. Four visual fields for each treatment condition were analyzed by fluorescence microscopy. Images of phalloidin staining were randomized for quantification of cell shape. Circularity of 15 cells in each image was analyzed using ImageJ software 1.54f, elipticity was analyzed using Imaris software (9.9.0, Bitplane). ZO‐1 staining was quantified in areas of cell‐cell contacts using Imaris software (9.9.0, Bitplane), ImageJ software (1.54f) and the labkit plugin.

### 2.7 Adhesion assay

Mesothelial cells (2.5‐3.5×10^4^/ 96 well) were grown in OCMI/5% FCS with or without stimulation (30 ng/ml rhIL‐17A, 1 ng/ml rhTNF, 30 ng/ml rhIL17A + 1 ng/ml rhTNF) on collagen‐I‐coated cell culture plates (5 µg/cm^2^; Gibco, Waltham, USA), and 2–3 days after seeding, medium and cytokines were replenished. Monolayer integrity was examined by microscopy of mesothelial cell stained with CellTracker Orange CMTMR (Invitrogen, Waltham, USA). After 4–5 days the mesothelial cell monolayer was washed three times with OCMI medium, CellTracker Green CMFDA (Invitrogen, Waltham, USA) labeled tumor cells were added and incubated for 6 h at 37°C. Thereafter, the monolayer was washed three times with OCMI medium to remove unattached tumor cells, and tumor cell adhesion was determined by microscopy of 7–8 visual fields per treatment condition. Tumor‐cell‐covered areas were quantified using ImageJ software (1.54f).

### 2.8 Murine omentum model

Mice were maintained and handled according to the approval by the local animal welfare officers. The protocol of Khan et al.^40^ was applied with some modifications.^41^ C57BL/6 mice were sacrificed by cervical dislocation and the omentum together with the pancreas and spleen were separated *en bloc* from the gastrointestinal tract, removed and placed in a beaker filled with ice‐cold PBS, where the pancreas and spleen remained at the bottom of the beaker, while the fat‐rich omentum floated at the top. The omentum was separated from the surrounding organs by trimming its base, and was fixed on a Millicell culture insert using Cell‐Tak Cell and Tissue Adhesive (Corning, #10317081). Cell‐Tak (7.5 µL) was evenly applied to the membrane of the insert and after complete drying, the membrane was washed twice with 1 mL sterile water. After drying, the membrane was placed in a 6‐well culture plate. In order to achieve optimal attachment of the omentum to the membrane, the omentum was spread on the pre‐treated membrane without medium prior to adding 3 mL of medium to the insert and 2 mL of medium to the surrounding well. Omenta were stimulated with 30 ng/mL rmIL‐17A (210‐17, Peprotech, Cranbury, USA) and 10 ng/mL rmTNF (12343014, ImmunoTools, Friesoythe, Germany). The culture was carried out under hypoxic conditions^42^ for 24 h at 37°C and 2% O2, 93% N_2_ and 5% CO_2_ as described.^41^ Whole mount staining of the omentum was performed based as published.^41^, ^43^ The following antibodies were used: anti‐CD45‐APC (559864, BD Biosciences, Franklin Lakes, USA); anti‐CD31‐PE (553373, BD Biosciences, Franklin Lakes, USA); anti‐ZO‐1‐AlexaFluor 488 (339188, Invitrogen, Waltham, USA); anti‐VCAM‐1‐V450 (105772, Biolegend, San Diego, USA). Fluorescence microscopy was performed on an Olympus FVMPE‐RS Multiphoton Microscope. Z‐stack images were taken from different regions of the omentum and image analysis was performed by machine‐learning‐based classification using Imaris software (9.9.0, Bitplane). In case of ZO‐1, analysis was restricted to areas of cell‐cell contacts.

### 2.9 Human CD4^+^ T‐cell isolation and differentiation

Peripheral blood mononuclear cells (PBMC) were isolated by Pancoll separating solution (PAN‐Biotech, Aidenbach, Germany) from buffy coats of healthy donors, kindly provided by the Center for Transfusion Medicine and Hemotherapy at Marburg University Hospital. CD4^+^ T cells were isolated from PBMCs by negative selection: the desired cell number PBMCs was incubated with FITC‐conjugated antibodies against CD8, CD15, CD16, CD19, CD36 and CD56 for 30 min at 4°C. Subsequently, cells were washed and incubated with anti‐FITC‐biotin/strepatividin complex for 15 min. Cells were washed again two times with sorting buffer (0.5% BSA, 2 mM EDTA in PBS) and incubated with biotin‐coupled beads on a rotator for 20 min. Non‐CD4^+^ T cells were retained in the magnetic field, the flow‐through fraction contained the CD4^+^ T cells with approximately 95% purity. One‐hundred thousand purified CD4^+^ cells per 96 well were stimulated with plate‐bound anti‐CD3 (ALX‐804‐822‐C100, clone TR66, Enzo Life Sciences, Farmingdale, USA) and anti‐CD28 (555725, clone CD28.2, BD Biosciences, Franklin Lakes, USA), and cultured in 200 µL of RPMI 1640 supplemented with 2 mM glutamine, 1 % (v/v) nonessential amino acids, 1 % (v/v) sodium pyruvate, penicillin (50 U/mL), streptomycin (50 mg/mL) and 10 % (v/v) FCS (Capricorn) or CM from mesothelial cells (untreated or treated with IL‐17A+TNF). Th17 differentiation in RPMI 1640 was performed in the presence of recombinant cytokines rhIL‐6 (200‐06), rhTGFβ (100‐21), IL‐23 (200‐23) and rhIL‐1β (200‐01B; all from Peprotech, Cranbury, USA). For intracellular cytokine analysis, cells were restimulated for 4 h with PMA (100 nmol/mL) and ionomycin (1 ng/mL) in the presence of brefeldin A (5 ng/mL). Cells were fixed with 2 % paraformaldehyde and permeabilized with 3 % saponin, stained with anti‐cytokine Abs specific for IL‐17A PE (512306, Biolegend, San Diego, USA), IFN‐γ APC (502512, Biolegend, San Diego, USA) and TNF PE‐Cy7 (502929, Biolegend, San Diego, USA) and analyzed with BD FACS Canto II and FlowJo software.

### 2.10 Single‐cell RNA sequencing (scRNA‐Seq) of patient‐derived mesothelial cells

Mesothelial‐cell‐enriched fractions obtained from the omenta of three different HGSC patients were analyzed by single‐cell RNA sequencing (scRNA‐Seq) using the Rhapsody single‐cell capture system (Becton, Dickinson and Company (BD). Targeted sequencing data, generated on an Illumina NextSeq 500j by the local Genomics Core Facility, was aligned and quantified using STARSolo,^44^ (version 2.7.10a) using the following parameters: soloType: CB_UMI_Complex, soloAdapterSequence: GTGANNNNNNNNNGACA, soloCBposition: [2‐9_2‐1, 2_4_2_12, 2_17_2_25], soloUMIposition: 3_10_3_17, soloCBmatchWLtype: EditDist_2, soloCellFilter: EmptyDrops_CR, outFilterScoreMinOverLread: 0, outFilterMatchNminOverLread: 0, outFilterMultimapScoreRange: 0, seedSearchStartLmax: 50, clip3pAdapterSeq: AAAAAAAAAAAAAAAAAAAAAAAAAAAAAAAAAAAAAA, outFilterMatchNmin: 25. The reference consisted of the Homo Sapiens genome sequence GRCH38.p13 retrieved together with the used gene models from Ensembl 108, extended with the Rhapsody sample tags for Homo sapiens. Cell barcodes were retrieved from.^45^, ^46^

The targeted gene set consisted of the Becton Dickinson Rhapsody Onco‐BC Targeted Panel (https://scomix.bd.com/hc/article_attachments/13766899704717) and a specifically selected panel to detect TNF+IL‐17A regulated genes in mesothelial cells based on our data described below in the Results section. The latter comprised the following gene sets (Table S3): (i) genes synergistically regulated by TNF and IL‐17A (Figure 3A), (ii) genes upregulated genes by IL‐17A+TNF with the highest expression, (iii) the epithelial‐mesenchymale transition (EMT) marker genes *CDH2*, *VEGF*, *SNAI1* and *ZEB1*, (v) all IL‐17 receptor genes and (vii) genes for cell type identification of other contaminating cell types^8^ (see below).

The STARsolo produced count matrix was processed with scanpy.^47^ Briefly, the gene set was reduced to the targeted gene set, which retained 97.5% of all uniquely identified molecules. The cells were filtered to retain those with 20 or more identified genes and 400 reads, and the genes were filtered to those with more than 21 observed cells, yielding a total of 11,290 cells. Sample source was assigned to cells based on sample tags. We required at least 5 sample tags per cell, and the 2nd most common sample tag for each cell to be present at less than 25% of the count of the most common one, to filter multiplets from different sources.

We performed UMAP^48^ and t‐SNE^49^, ^50^ based embedding and read counts were normalized to 10k and log‐transformed. Cells were clustered using Louvain^51^ clustering, and initially annotated to cell types using SCSA.^52^ The mesothelial cell cluster of interest was identified by expression of the mesothelial markers genes *ITLN1* (Intelectin 1)*, HP* (Haptoglobin) or *UPK3B* (Uroplakin 3B) (see Human Protein Atlas; https://www.proteinatlas.org/humanproteome/single+cell+type). This cluster was further filtered to not contain cells showing expression of any of the following cell‐type‐selective markers (Human Protein Atlas and ref.^8^) expressed in the other clusters: epithelial (tumor) cells: *CLDN4, EPCAM*; macrophages: *FCER1G, LYZ*; T cells: *CD3E, GZMB*; B cells: I*GHM, JCHAIN*; adipocytes: *JCAD, S1PR1, VWF*. The mesothelial cells were then subjected to UMAP and Louvain clustering and quantified according to the expression of the IL‐17A+TNF‐induced genes identified by bulk RNA‐Seq (Table S4).

### 2.11 Bulk RNA‐Seq of mesothelial cells

Bulk RNA‐Seq was performed by Novogene (UK) using a proprietary library generation protocol. Paired end RNA‐Seq reads were aligned using STAR^44^ (version 2.7.10a) against Ensembl 104, using only the R1 read. Reads were quantified within the exons of protein coding transcripts and normalized to ‘tags per million’. Differential expression was estimated using EdgeR^53^ and filtered (at least 15 reads, FDR < = 0.05, | log2FC| > = 1). Pathway analyses were conducted using enrichr and the Molecular Signatures Database (MSigDB) hallmark gene set collection.^54^

### 2.12 Affinity proteomics of CM from mesothelial cells

Targeted proteomics analysis (proximity extension assay technology; PEA) was performed using the Olink Explore 3072 platform, following the Olink standard protocol (v1.5, 2022‐12.21). All samples were randomized and plated on a 96‐well plate and processed in one batch. The generated libraries were analyzed by next‐generation sequencing (NGS) and data were evaluated to yield Normalized Protein eXpression values (NPX; Olink‐provided arbitrary unit in log_2_ scale) as described.^13^ If mediators were included in more than one panel (CXCL8, IDO1, IL6, LMOD1, SCRIB, TNF) mean NPX values were used in subsequent analyses. Cytokine‐regulated proteins were identified by determining the difference of the respective NPX values. The extent of regulation was calculated as 2^∆NPX^ (fold change; FC) and results were expressed as the median of n = 5 biological replicates. Nominal p values determined by unpaired t test were adjusted for multiple testing by posthoc analysis (Benjamini‐Hochberg method) to control the false discovery rate (FDR).

### 2.13 Data deposition

RNA‐seq data was anonymized using BAMBoozle and deposited at EBI Array Express under accession number E‐MTAB‐13497. Single cell sequencing was processed as described, and the resulting expression matrix deposited at EBI Array Express under accession number E‐MTAB‐13498.

### 2.14 Statistical analyses

Statistical analyses were performed using GraphPad Prism v9.4. Our data presentation includes bar graphs (mean ± s.d), jitter plots (mean ± s.d.) and boxplots (median, quartiles). Each data point represents one biological replicate unless otherwise stated. To assess normality of distribution and homogeneity of variances we utilized Shapiro‐Wilk and Brown‐Forsythe test, respectively, for all datasets. Comparisons between two groups were carried out using two‐tailed unpaired t‐tests or two‐tailed paired t‐test as appropriate. The confidence interval chosen for all test was 95%. For comparisons involving multiple conditions, we performed one‐way or two‐way analysis of variance (ANOVA) followed by a Dunnett's multiple comparisons test. In cases datasets did not conform to normality criteria, non‐parametric Kruskal‐Wallis test was employed, followed by Dunn's multiple comparisons test. A critical value for significance of P < 0.05 (*) was used throughout the study. Statistical thresholds of P < 0.01 (**), P < 0.001 (***) and P < 0.0001 (****) are indicated in the figures by asterisks. Associations with relapse‐free survival (logrank test), hazard ratio (HR) and median survival times were analyzed using the Python Lifelines KaplanMeierFitter and CoxPHFitter functions.

## 3 RESULTS

### 3.1 Th17 cells are detectable in early omental metastases

**FIGURE 1:**
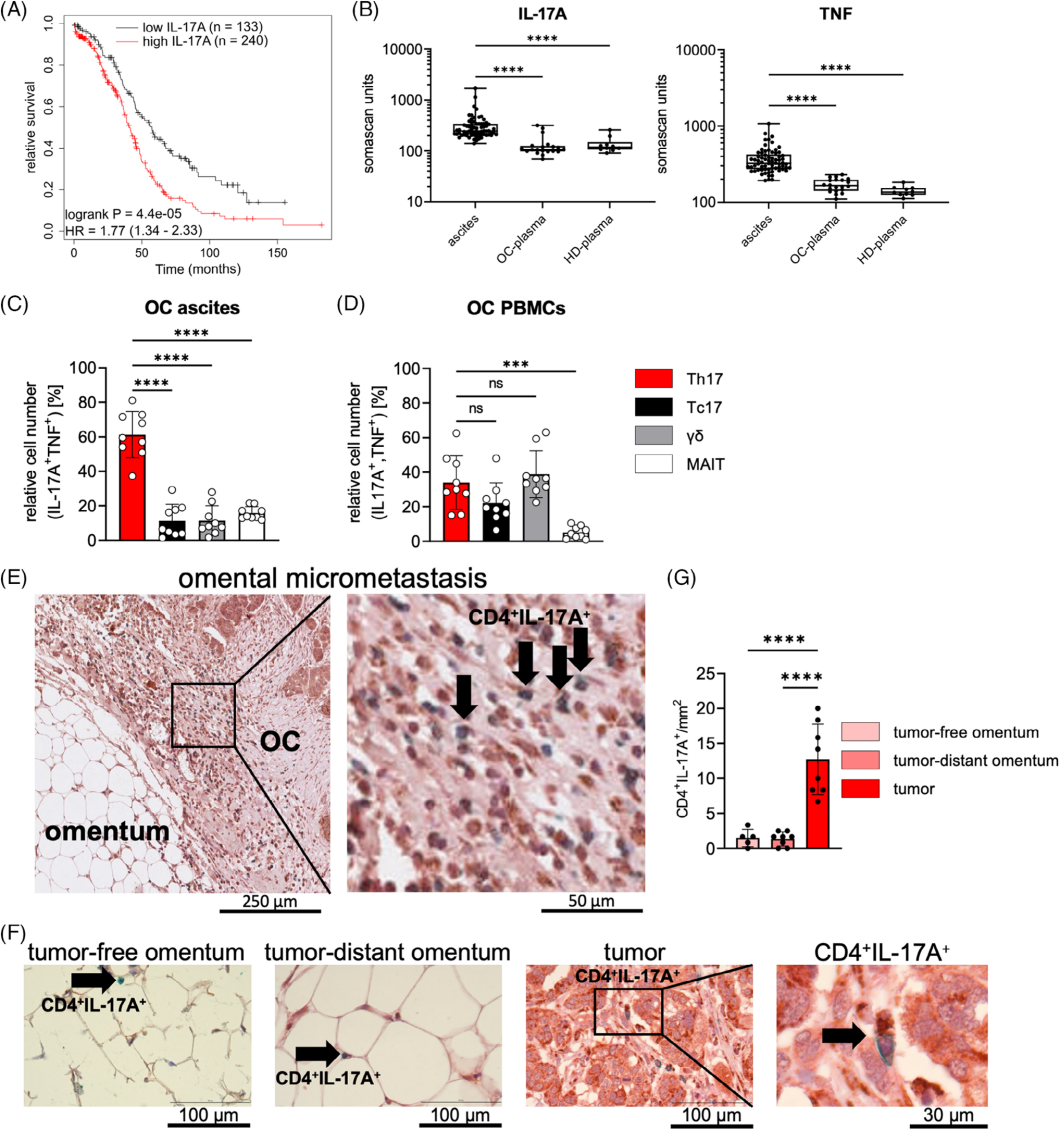
Clinical associations of Th17 cells with OC. (A) Kaplan‐Meier curves showing the association of *IL17A* gene expression with overall survival of OC. The analysis was performed using the KM‐Plotter database^55^ with auto‐selected cut‐offs for n = 373 patients (RNA‐Seq data for OC in the pan‐cancer dataset). HR: hazard ratio. (B) SOMAscan signals for IL‐17A and TNF in OC ascites (n = 70), matched plasma samples from OC‐patients (OC‐plasma; n = 20) and plasma from healthy donors (HD‐plasma; n = 10). Boxplots show the mean, minimum, maximum and quantiles; data points represent different patients. ****p<0.0001 determined by Kruskal‐Wallis test. (C) Quantification by flow cytometry of IL‐17A^+^/TNF^+^ double‐positive cells in CD14‐depleted OC ascites cells (n = 9) among CD4^+^, CD8^+^, γδ, and MAIT cells. (D) Analysis as in panel C for matched OC PBMCs (n = 9). (E) Representative immunostaining of an omental micrometastasis for CD4 (green) and IL‐17A (brown). (F) Representative pictures showing immunostaining of CD4 (green) and IL‐17A (brown) in tumor‐free omentum, tumor‐distant omentum and tumor tissue in omental micrometastases. Arrows indicate CD4^+^/IL‐17A^+^ double‐positive cells. (G) Quantification of CD4^+^/IL‐17A^+^ double‐positive cells in tumor‐free omentum (n = 5), tumor‐distant omentum (n = 8) and tumor tissue (n = 8). Bar plots in panels C, D and G indicate the mean±SD of biological replicates. ***p<0.001, ****p<0.0001 determined by one‐way ANOVA followed by Dunnett's multiple comparison test. ns: not significant.

Considering the controversial role of IL‐17A in OC, we analyzed public RNA‐Seq data for OC patients (n = 374) for potential correlations of *IL17A* gene expression with clinical outcome. As shown in Figure 1A, interrogation of the Kaplan‐Meier plotter database^55^ identified a significant (p = 0.00004; FDR = 0.01) inverse association of *IL17A* expression with overall survival, suggesting a pro‐tumorigenic role (Figure 1A). This finding is consistent with IL‐17A levels in ascites determined in a previous study using affinity proteomics (SOMAscan),^56^ which showed significantly elevated levels of IL‐17A in OC ascites compared to plasma from OC patients or healthy donors (Figure 1B). Likewise, TNF concentrations in ascites were significantly increased (Figure 1B). Furthermore, the ascites level of the soluble receptor subunit IL‐17RC measured by SOMAscan was significantly associated with a short relapse‐free survival (Figure S1A). Abundant soluble IL‐17RC is produced by shedding and therefore presumably reflects high levels of the functional membrane‐bound receptor, which would support the hypothesis that IL‐17 signaling is linked to OC progression.

To detect the main cellular source of IL‐17A and TNF in ascites, we reanalyzed our published RNA‐Seq data and identified tumor‐associated T cells (TAT) as the cell type with the strongest expression of both *IL17A* and *TNF* (Figure S1B andC). We therefore stained the monocyte‐ and macrophage‐depleted cellular fraction of ascites for specific markers of IL‐17A producing T cells, i.e., Th17, Tc17, γδ 17 T and mucosal‐associated invariant (MAIT) cells, as well as for IL‐17A and TNF (Figure S2A andB). Intriguingly, approximately 80% of all IL‐17A‐producing T cell subtypes were double‐positive for IL‐17A and TNF, in both ascites and peripheral blood (Figure S2C). Among those, Th17 cells were the most prevalent population, constituting approximately 60% of the IL‐17A^+^TNF^+^ double‐positive cell population in ascites (Figure 1C). In contrast, the relative number of Th17 cells in peripheral blood of OC‐patients was lower (approx. 30%) as compared to ascites and similar to the abundance of Tc17 and γδ 17 T cells (Figure 1D). These data indicate an enrichment of IL‐17A^+^TNF^+^ double‐positive Th17 cells in OC‐ascites.

Since IL‐17A and TNF were previously reported to impact the activation state of mesothelial cells,^26^ we investigated whether Th17 cells are present in the vicinity of mesothelial cells at sites of omental micrometastases.^5^, ^7^ Immunostaining of tissue sections from OC‐omental micrometastases, tumor‐free and tumor‐distant omentum for CD4^+^ and IL‐17A^+^ double‐positive cells revealed a significant increase in CD4^+^IL‐17A^+^ Th17 cells in omental micrometastases (Figure 1E‐G). We validated the staining using a second marker of Th17 cells, the transcriptional regulator of Th17 cells, RORγt.^21^, ^57^ Similarly, CD4^+^RORγt^+^ Th17 cells accumulated at the metastatic sites (Figure S3A‐C). Based on these observations we investigated the hypothesis that Th17 cells promote metastasis‐associated processes by interacting with mesothelial cells.

### 3.2 IL‐17A and TNF alter the transcriptome of mesothelial cells towards an inflammatory and mesenchymal phenotype

**FIGURE 2:**
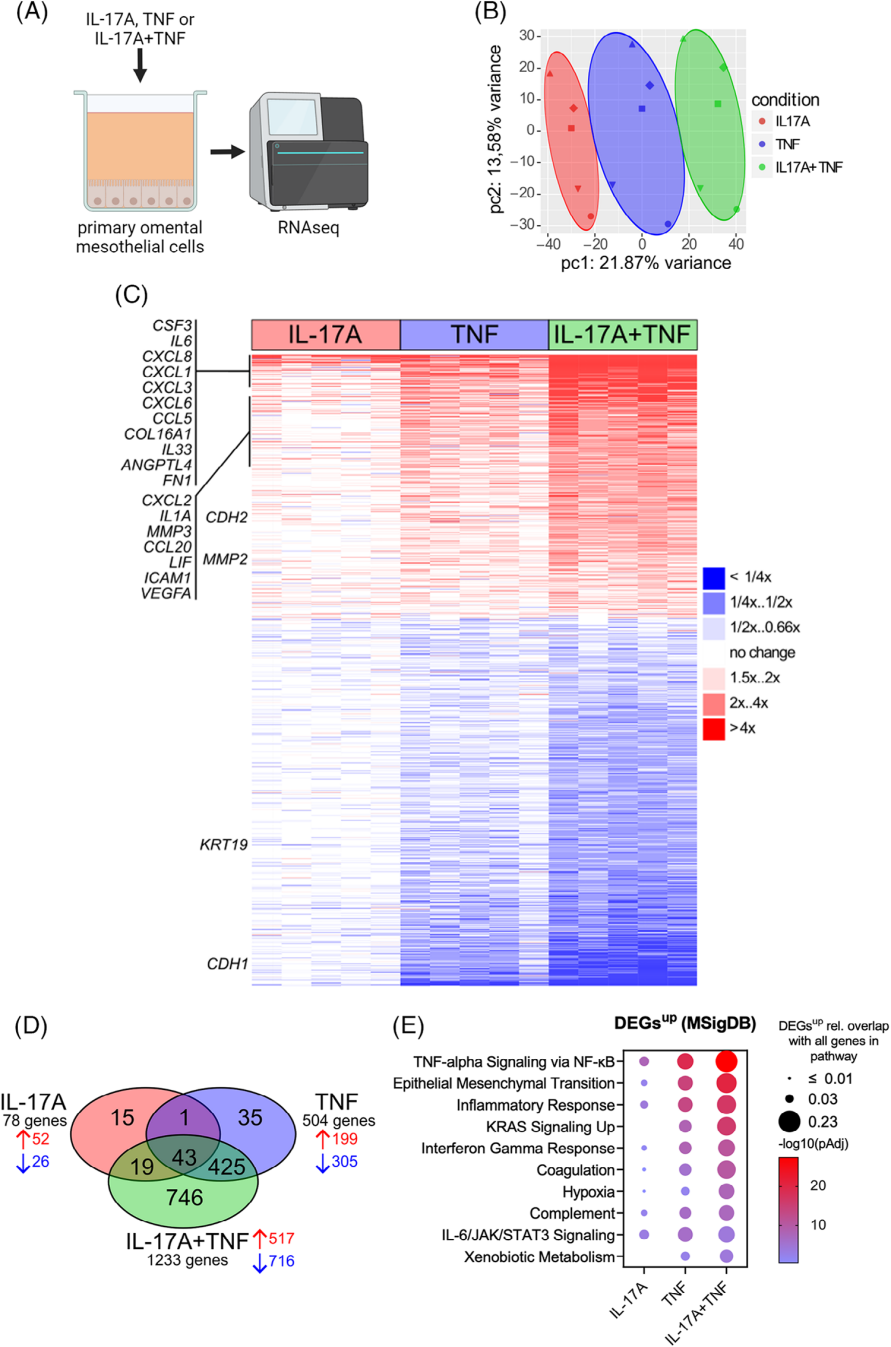
IL‐17A and TNF alter the transcriptome of mesothelial cells towards an inflammatory and mesenchymal phenotype. (A) Mesothelial cells from 5 patients were treated for 48 h with rhIL‐17A, rhTNF or both cytokines prior to bulk RNA‐Seq analysis. (B) Principal component analysis of RNA‐Seq data from mesothelial cells treated as described in (A). Colors: treatment conditions; shapes: patients (n = 5); colored circles: manually added highlights of groups separated by conditions (pc1). (C) Heatmap of differential gene expression (FC relative to untreated cells; n = 5). (D) Venn diagram showing the numbers of differentially regulated genes for different treatment conditions. (E) Molecular Signature (MSigDB) hallmark pathway analysis.^54^ The bubble graph displays the 10 most significantly enriched pathways for each treatment and the relative overlap with all genes of the respective pathway.

Since Th17 cells act via secreted cytokines,^21^, ^57^ and in OC ascites, Th17 cells were mostly double‐positive for IL‐17A and TNF, we next analyzed the impact of IL‐17A, TNF or their combination on the transcriptome of omental mesothelial cells from five different OC patients. Surface expression of IL‐17RA and IL‐17RC, the receptors necessary for IL‐17A signaling, was validated in omental mesothelial cells from OC patients (Figure S4). RNA sequencing analysis revealed minor changes in the gene expression caused by IL‐17A alone, while the impact of TNF was more pronounced. The strongest changes in the transcriptome were induced by a combined treatment with IL‐17A and TNF (Table S4), as visualized by the principal component analysis (PCA) biplot, the heatmap, as well as the Venn diagram of differentially regulated genes (n = 1284, |log2FC| > 1, FDR>0.05) in Figure 2A‐D. While only a small number of genes were exclusively regulated by single treatment with IL‐17A (n = 15) or TNF (n = 35) alone, the vast majority of differentially regulated genes (62.3%; n = 746 of 1284) was regulated only by the combination of IL‐17A and TNF (Figure 2D).

Consistent with these findings, functional analysis revealed that the combined treatment with IL‐17A and TNF had the strongest impact on the regulation of genes involved in TNF signaling via NF‐κB (*CXCL1, CXCL2, CXCL3, CXCL6, ICAM1, IL6, EGR1, LIF, IL1A, VEGFA*), EMT (*COL16A1, CXCL8, CDH2, FN1, MMP2, MMP3*), inflammatory response (*CSF3, CCL20, CXCL8*), KRAS signaling (*IL33, ANGPTL4, DUSP6*)) and IFNγ‐response (*TNFAIP6, TNFAIP3, CCL5, IRF8, OAS2, GBP4*) (Figure 2E; Table S5). This indicates that treatment with a combination of the IL‐17A and TNF induces a unique transcriptional state in omental mesothelial cells, eliciting reprogramming towards a mesenchymal and proinflammatory phenotype. Interestingly, the induction of numerous chemokines and cytokine genes (*CXCL1, CXCL2, CXCL3, CXCL6, IL6, LIF, IL1A, CSF2, CCL20, CXCL8, IL33, CCL5)* as well as genes involved in the regulation of cell‐cell contact and tissue remodeling (*COL16A1, CDH2, FN1, MMP2, MMP3, ANGPTL4*) represented characteristic features of this transcriptional state, indicating a possible involvement in the recruitment and regulation of other cells in the OC TME.

### 3.3 IL‐17A and TNF synergistically induce genes linked to mesenchymal transition of mesothelial cells

**FIGURE 3:**
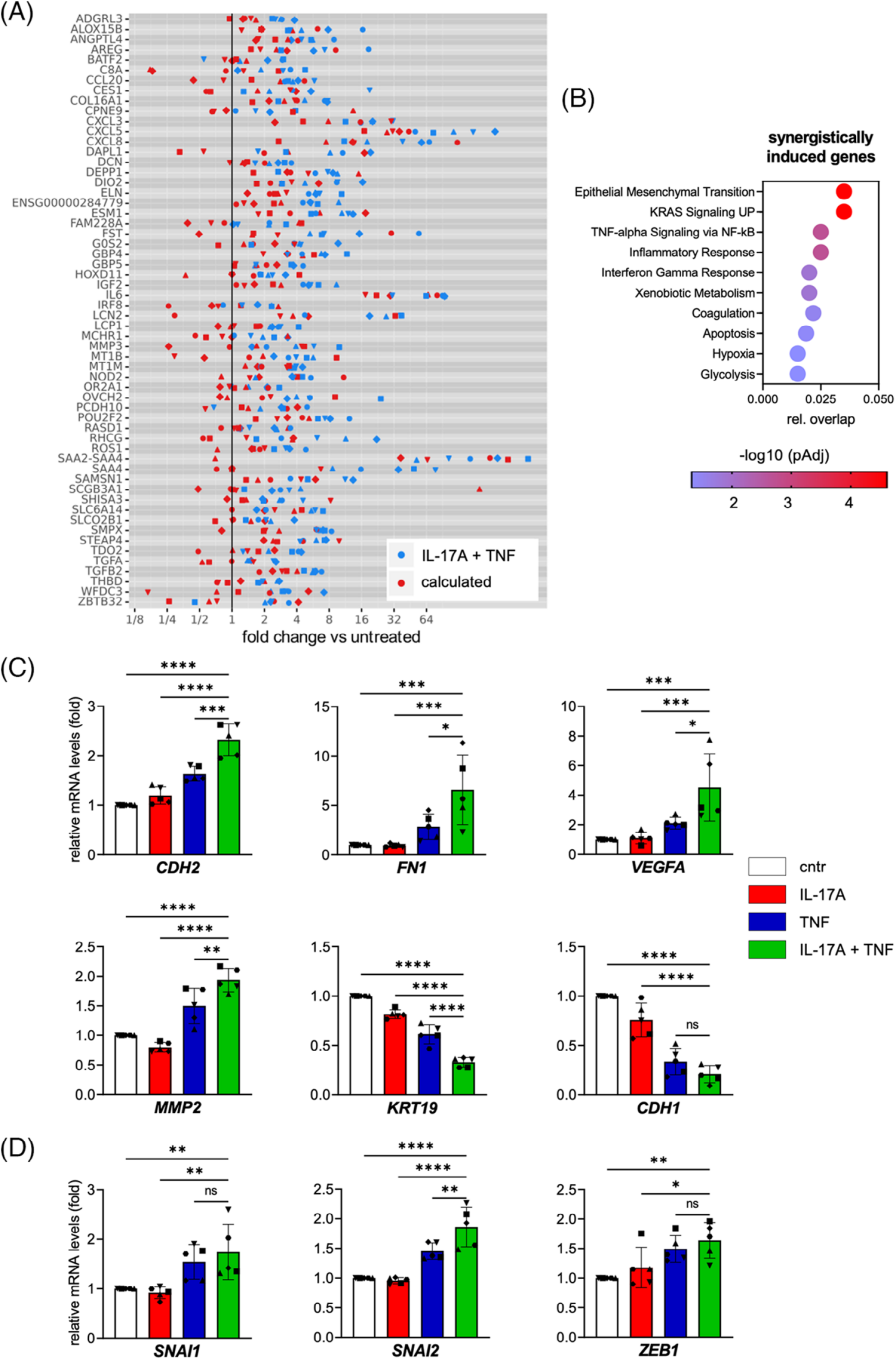
IL‐17A and TNF synergistically induce genes involved in mesenchymal transition of mesothelial cells. (A) Synergistically induced genes of mesothelial cells treated as in Figure 2 (n = 5 patients). Jitter plot of the calculated additive and the actually observed induction (FC) by TNF+IL‐17A treatment. The plot shows genes with a ≥2‐fold ratio (= 1 log2 unit) of observed/expected median fold change. (B) Molecular Signature (MSigDB) hallmark pathway analysis.^54^ The bubble graph presents the 10 most significantly enriched pathways of synergistic candidate genes defined in (A) and the relative overlap with all genes of the respective pathway.(C) RT‐qPCR of EMT marker gene expression after treatment of mesothelial cells for 48 h with rhIL‐17A, rhTNF or both cytokines. Expression values were normalized to the untreated control (cntr). (D) qPCR analysis of selected EMT‐transcription factor genes performed as in panel B. Bar plots show the mean±SD of biological replicates (n = 5). *p<0.05, **p<0.01, ***p<0.001, ****p<0.0001 were determined by by one‐way ANOVA followed by Dunnett's multiple comparison test; ns: not significant.

Next, we addressed the question whether IL‐17A and TNF act synergistically to regulate the transcriptome and the pathways identified above. To this end, we compared the added FC values for individual IL‐17A and TNF treatments to the FC measured for the combined treatment. Values at least one log2 unit higher than the calculated additive FC were designated synergistic (Figure 3A). Using this approach, we identified 58 synergistically regulated genes after treatment with IL‐17A and TNF in mesothelial cells (Figure 3A). Pathway analysis of these synergistically regulated genes identified EMT as the most prominent pathway, followed by KRAS signaling, TNF signaling via NF‐κB and inflammatory response (Figure 3B). These analyses suggest that the mesenchymal reprogramming of mesothelial cells is primarily regulated by the synergistic action of IL‐17A and TNF.

To investigate the mesenchymal transition of mesothelial cells driven by the combination of IL‐17A and TNF in detail, we analyzed the regulation of the classical EMT marker genes.^58^, ^59^, ^60^, ^61^ As shown by RT‐qPCR (Figure 3C), IL‐17A and TNF induced the mesenchymal markers *CDH2*, *FN1*, *VEGFA* and *MMP2*, while the epithelial markers *KRT19* and *CDH1* were repressed. The combined treatment with IL‐17A and TNF yielded the strongest effect (Figure 3C), supporting the conclusion that IL‐17A and TNF cooperate to regulate MMT. Synergistic effects on these typical EMT marker genes were, however, not observed, indicating a specific program of IL‐17A/TNF‐induced MMT distinguishing it from classical EMT. This conclusion is supported by the observation that the EMT‐driving transcription factors *SNAI1*, *SNAI2* and *ZEB1* ^61^ were induced by TNF, which was only slightly enhanced by combining both cytokines, if at all (Figure 3D).

A conspicuous feature of the IL‐17A+TNF‐regulated transcriptome (Table S4) is the up‐ or downregulation of multiple genes coding for neurotransmitter receptors. These include the acetylcholine receptor subunit CHRNB2, the adrenergic receptor ADRB2, the γ‐aminobutyric acid receptor subunits GABRE and GABRA5, the histamine receptor HRH1 and the serotonin receptors HTR2A and HTR2B. It is, therefore, conceivable, that neurotransmitters contribute to the reprogramming of mesothelial cells, which may be of interest considering the emerging role of these molecules in cancer metastasis.^62^, ^63^

### 3.4 The IL‐17A+TNF‐induced transcriptome can be identified in a subset of mesothelial cells in the omentum of OC patients

**FIGURE 4:**
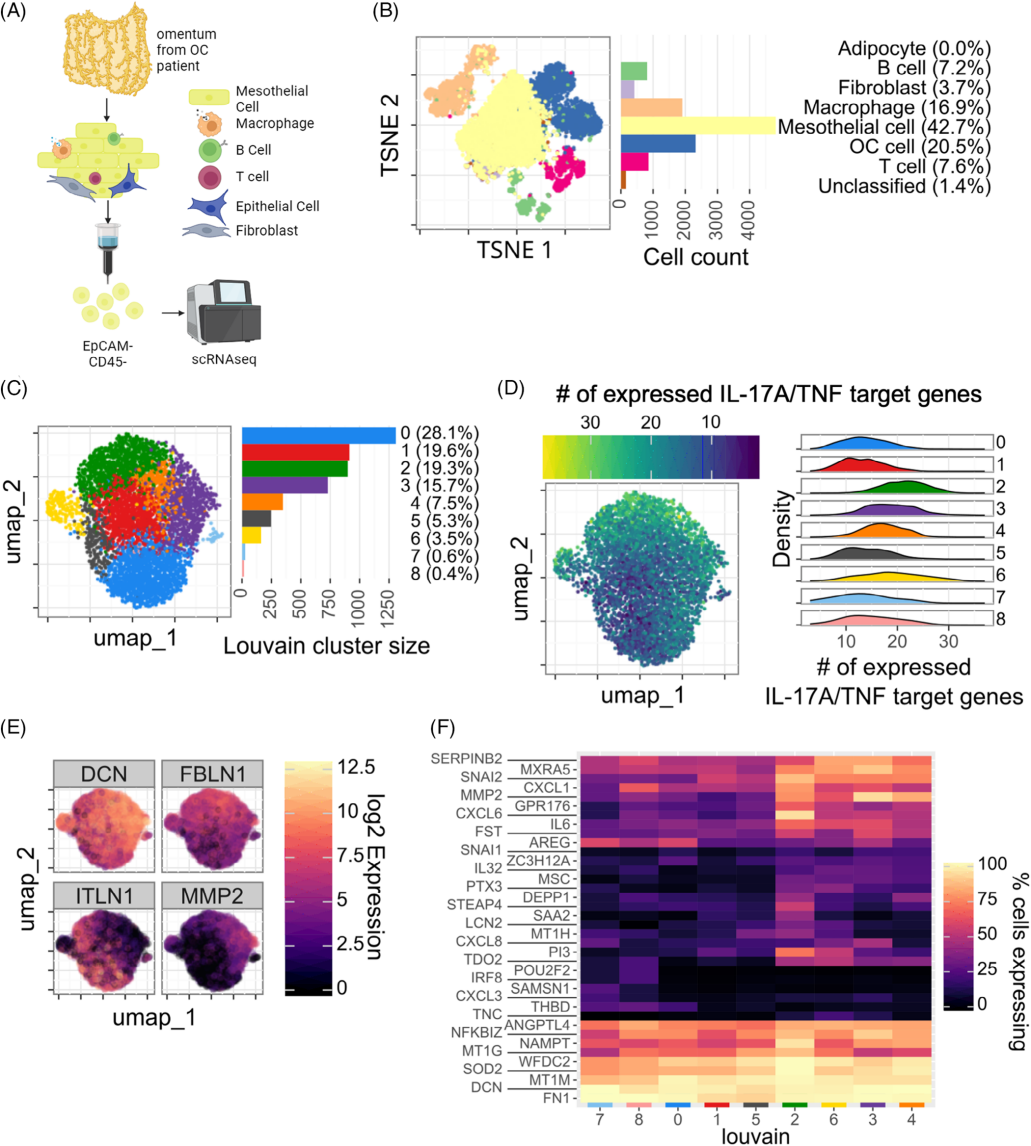
IL‐17A‐ and TNF‐induced transcriptional changes can be identified in a subset of mesothelial cells from OC patients. (A) Mesothelial cells were enriched from tumor‐free omentum by MACS depletion of EpCAM^+^ and CD45^+^ cells prior to targeted scRNA‐Seq. (B) Two‐dimensional embedding of scRNA‐Seq data by t‐SNE. Cells were clustered via Louvain and annotated to cell type using the marker genes listed in the Methods and Materials section. The bar chart shows distribution of cell types in the enriched cell population (see also Figure S5). (C) Two‐dimensional embedding (UMAP) and Louvain clustering (filtered as described in Methods and Materials) of mesothelial cells identified in panel B. (D) Number IL‐17A/TNF‐induced genes expressed in different clusters. Across all clusters, expression of 67 genes was detectable out of 85 genes included in the scRNA‐Seq analysis. Embedding was performed as in panel C. (E) Visualization of fibroblast *(DCN, FBLN1, MMP2)* and mesothelial *(ITLN1)* marker gene expression in the Louvain clusters identified in panel C. (F) Heatmap of IL‐17A+TNF induced genes expressed in Louvain clusters from panel C (filtered for genes expressed in at least 20% of the cells in at least one cluster).

We next sought to assess whether the findings obtained from RNA‐Seq from in vitro IL‐17A‐ and TNF‐treated mesothelial cells replicate in vivo. To this end, we analyzed mesothelial‐cell‐enriched (EpCAM‐ and CD45‐negative) fractions from tumor‐free omentum regions of three different OC‐patients by targeted single‐cell RNA sequencing (scRNA‐Seq) (Figure 4A), focusing on IL‐17A and TNF regulated genes combined with genes regulated in malignancy (Onco‐BC), as well as markers distinguishing different cell populations (Table S3). By applying t‐SNE‐based embedding and screening for cell‐type‐selective markers (see Methods and Materials) we identified mesothelial cells as the major population (42.7 %), but also epithelial cells (presumably tumor cells), macrophages, B‐cells, T cells and fibroblasts (Figures 4B and S5A). Cells expressing the mesothelial marker genes *ITLN2, HP* or *UPK3B* were classified as mesothelial cells. As shown in Figure S5B, the majority of these cells also expressed fibroblast marker genes to varying degrees with a moderate negative correlation between mesothelial and fibroblast marker, suggesting that this cell population represents a continuum of mesothelial cells at different stages of mesenchymal transition. This finding also supports the hypothesis that MMT occurs in the mesothelium of OC patients.

Further analysis of this population identified a total of 9 Louvain clusters of cells (Figure 4C). Across all clusters, expression of n = 465 genes (out of 516 analyzed) was detectable by scRNA‐Seq (Table S3), among which n = 108 represent IL‐17A+TNF‐induced genes (out of 130 analyzed). As depicted in Figure 4D, the 9 clusters differed considerably in the number of detectable cells with IL‐17A/TNF‐induced genes, with clusters 2, 3, 4 and 6 harboring the highest frequency of such cells and clusters 0, 1, 5, 7 and 8 presenting with the lowest numbers. Intriguingly, the clusters with high numbers of cell expressing IL‐17A/TNF‐induced genes displayed the highest expression of the fibroblast markers *DNC, FBLN1* and *MMP2*, concomitantly with a lower expression of the mesothelial marker *ITLN1* (Figure 4E). These observations suggest that a subset of patient‐derived omental mesothelial cells expressing IL‐17A/TNF‐induced genes display a shift towards a mesenchymal phenotype. This conclusion is supported by a closer look at the cluster‐related expression of IL‐17A/TNF‐induced genes (Figure 4F). This analysis revealed similarities in the expression of MMT‐ or inflammation‐associated genes (e.g., *FN1, DCN, NFKBIZ, ANGPTL4*) but also specific patterns characterized by a high expression in clusters 2, 3, 4 and 6, including *AREG, IL6, CXCL1, CXCL6, MMP2, SNAI1* and *SERPINEB2*, suggesting both common and subset‐specific pathways of mesenchymal reprogramming. Finally, embedding analyses as in Figure 4C performed for individual patients revealed both common and patient‐specific contributions to the observed clustering of IL‐17A/TNF‐induced genes (Figure S6).

### 3.5 IL‐17A and TNF disturb mesothelial monolayer integrity and facilitate adhesion of tumor cells

**FIGURE 5:**
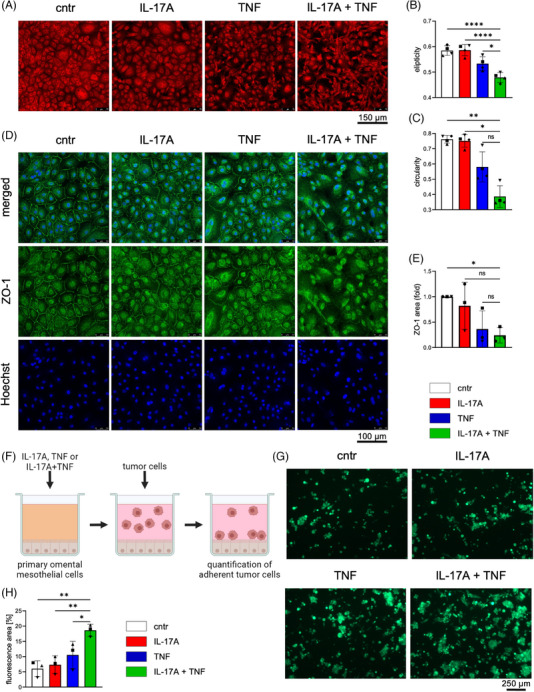
IL‐17A and TNF perturb mesothelial monolayer integrity and promote adhesion of tumor cells. (A) Phalloidin staining of mesothelial cells treated with rhIL‐17A, rhTNF or both cytokines for 96 h. Medium and treatment were renewed after 48 h. Representative pictures are shown. (B, C) Quantification of ellipticity (B; Imaris software) and sphericity (C; ImageJ software) of experiments shown in panel A. Four random images were analyzed for each treatment condition and n = 4 patients. (D) ZO‐1 (green) and Hoechst (blue) staining of mesothelial cells treated as in panel A. The pictures are representative of three experiments with cells from different patients. (E) Quantification of ZO‐1 area at cell‐cell contacts with ImageJ software and the Labkit plugin. Three random images for each treatment condition were analyzed and n = 3 patients. (F) Schematic overview of the experimental design of the tumor cell adhesion assay. Mesothelial cells were seeded on 96‐well plates coated with collagen I, stimulated with rhIL‐17, rhTNF or both cytokines, and cultured for 4–5 days till formation of a dense monolayer. Subsequently, wells were washed and CellTracker Green‐stained tumor cells were added for 6 h. (G) Pictures of adherent OC‐tumor cells treated as described in panel F. Representative images are shown. (H) Quantification of adhesion assays as in panel G. Attached tumor cells were quantified by measuring the relative area covered by fluorescently labeled tumor cells using ImageJ software (n = 3 patients). The bar plots show the mean ±SD for biological replicates.*p<0.05, **p<0.01, ****p<0.0001 were determined in panels B, E and H by one‐way ANOVA followed by Dunnett's multiple comparison test, in panel C by Kruskal‐Wallis test followed by Dunn's multiple comparison test: ns: not significant.

Mesenchymal reprogramming of mesothelial cells is characterized by loss of cell‐cell junctions, cytoskeleton reorganization, disappearance of apical and basal cell polarity as well as the acquisition of a fibroblast‐like migratory phenotype.^58^, ^59^, ^60^ To investigate whether mesothelial cells undergo such phenotypical alterations following treatment with IL‐17A and TNF, we first studied the effect of these cytokines on mesothelial cell morphology. Phalloidin staining of OC‐patient‐derived mesothelial cells cultured with IL‐17A alone showed no difference in monolayer density or cell morphology, whereas both slightly differed after treatment with TNF. Consistent with the observed effects on the transcriptome, the combined treatment with IL‐17A and TNF resulted in a drastic change of the cell morphology towards a fibroblast‐like phenotype with elongated cell body and long cytoplasmic extensions (Figure 5A‐C). These changes were also observed by bright‐field microscopy, which additionally revealed small gaps in the monolayer of IL‐17A+TNF‐treated cells (Figure S7A).

Junctional complexes, including tight junctions, gap junctions and desmosomes are hallmarks of mesothelial cell monolayers.^59^, ^60^ To address the question how IL‐17A and TNF treatment influences mesothelial‐cell monolayer integrity, we focused on the tight‐junction‐associated scaffold protein zona occludens‐1 (ZO‐1). ZO‐1 is an intracellular plaque protein that localizes mainly to cell‐cell adhesion membrane complexes and forms a scaffold connecting transmembrane proteins and the actin cytoskeleton. During mesenchymal transition, ZO‐1 relocalizes from membrane complexes, accumulates in the cytoplasm, and eventually translocates to the nucleus.^64^ As detected by immunofluorescence microscopy, IL‐17A had no effect and TNF led to only slight changes in the intracellular distribution of ZO‐1, while the combined treatment triggered a clear loss of ZO‐1 localization to the cell‐cell boundaries (Figure 5D , 5E) in the absence of detectable changes in nuclear localization (Figure S7B). Furthermore, increased cytoplasmic occurrence of ZO1 was accompanied by strong morphological changes of mesothelial cells (Figure 5D , 5E). These results further support the notion that IL‐17A and TNF cooperate to induce MMT.

The altered expression of ZO‐1 was further confirmed in an *ex vivo* setting, with murine omenta cultured in the presence of IL‐17A and TNF. As shown in Figure S8A andB, a significant loss of ZO‐1 staining at mesothelial cell‐cell contacts was observed following 24 h of treatment. Furthermore, staining of mesothelial cells for the MMT marker VCAM‐1 was significantly increased (Figure S8A andB). This is consistent with scRNA‐Seq data obtained with patient‐derived material, which revealed an increased number of *VCAM1*‐positive cells in clusters enriched for elevated expression of the mesothelial marker gene *MMP2* (Figure S9; >50% positive cells in Louvain clusters 2, 3, 4 and 6) as well as IL‐17A/TNF‐induced genes (Figure 4D). VCAM‐1 expression was previously shown to be upregulated on mesothelial cells by TNF treatment, thereby enabling cancer‐mesothelial cell interactions leading to OC cell attachment and invasion.^16^ We thus investigated whether IL‐17A influences the TNF‐driven adhesion of cancer cells to a mesothelial monolayer in vitro (Figure 5F). Pretreatment of a mesothelial cell monolayer with TNF enhanced the adhesion of tumor cells, with clear further enhancement by combined TNF/IL‐17A treatment (Figure 5G, H).

In summary, these data indicate that IL‐17A and TNF cooperatively reprogram mesothelial cells towards a mesenchymal phenotype, resulting in a loss of monolayer integrity and increased tumor cell attachment.

### 3.6 IL‐17 and TNF synergistically direct the secretome of mesothelial cells towards a Th17‐promoting environment

**FIGURE 6:**
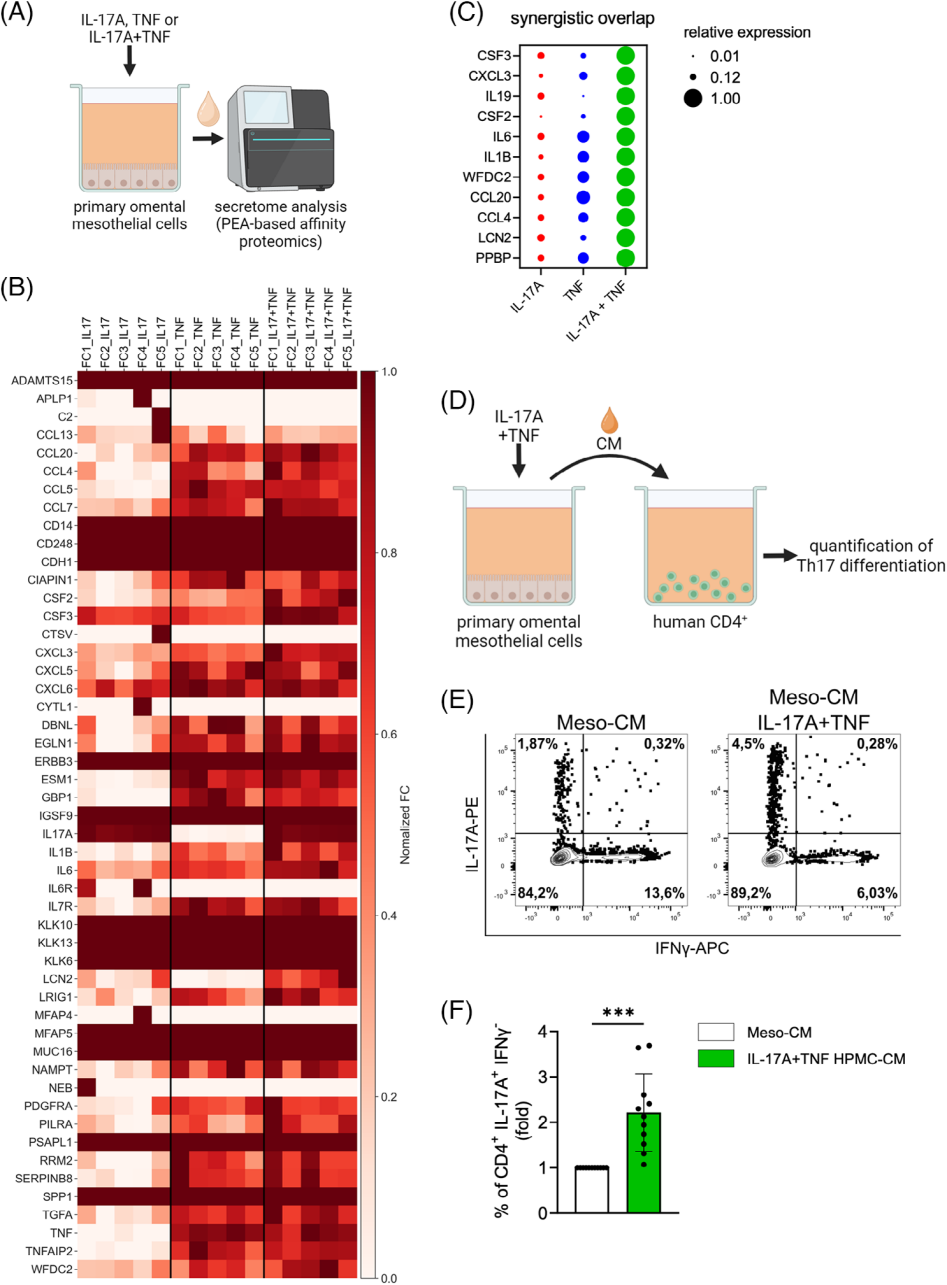
IL‐17A and TNF synergistically direct the secretome of mesothelial cells towards a Th17‐promoting environment. (A) Affinity proteomics analysis of the mesothelial cell secretome after 48 h treatment with rhIL‐17A, rhTNF or both cytokines. (B) Heatmap showing the top 50 significantly upregulated proteins after combined treatment (p <0.05 by paired t test; synergism defined as FC of combined treatment > added individual FC values x 1.5). FC values were protein‐wise normalized. (C) Bubble graph of synergistically induced proteins. Relative expression normalized to combined IL‐17A/TNF treatment is shown. (D) Schematic overview of the experimental design. CM from mesothelial cells treated with rhIL‐17A and rhTNF and from untreated cells were collected after 24 h. CD4^+^ cells from PBMCs were treated with CM for 8 days. Induction of Th17 was measured by flow cytometry. (E) Representative staining of CD4^+^ cells after treatment with CM from untreated compared to IL‐17A/TNF‐treated mesothelial cells. (F) Increase (FC) of IL‐17A^+^/IFNγ^−^ cell frequency after treatment as in panel E. Bars show the mean ±SD of n = 11 biological replicates. ***p<0.001 was determined by two‐tailed unpaired t‐test.

The RNA‐Seq data described above revealed a strong upregulation of chemokine and cytokine genes by IL‐17A and TNF (Figure 2C), which we hypothesized to promote the attraction of immune cells, as well as influence their activation and differentiation state. We therefore evaluated the influence of IL‐17A and TNF on the protein secretion profile of mesothelial cells by PEA‐based affinity proteomics^65^, ^66^ targeting 2923 proteins (Figure 6A; see Material and Methods and Materials for details). As shown in Figure 6B and Table S6, IL‐17A only weakly regulated the secretome of mesothelial cells, while TNF had a strong effect that was further enhanced by IL‐17A co‐treatment. For 11 proteins we found synergistic regulation by IL‐17A and TNF (Figure 6C). Among the synergistically regulated proteins were G‐CSF (CSF3), a known target of IL‐17A in mesothelial cells,^34^ as well as the OC biomarker WFDC2 (HE4).^67^, ^68^

The upregulated proteins include IL‐6, IL‐1β and CCL20, consistent with their increased abundance in ascites found in our previously published SOMAscan dataset^56^ (Figure S10). IL‐6 and IL‐1β, either alone or cooperatively with low concentrations of TGFβ and/or IL‐23, drive CD4^+^ lymphocyte differentiation into the Th17 phenotype.^21^, ^57^, ^69^, ^70^, ^71^ In OC, TGFβ family members are secreted by different cell types,^8^ including mesothelial cells (Table S6), and are present in ascites.^56^ Considering the increased accumulation of Th17 cells in the early omental metastases and the induction of IL‐6 and IL‐1β by IL‐17A/TNF‐triggered mesenchymal reprogramming, we speculated the secretome of reprogrammed mesothelial cells to promote differentiation of CD4^+^ T cells towards a Th17 fate. To test this hypothesis, we stimulated CD4^+^ T cells obtained from peripheral blood of healthy donors through T‐cell receptor (TCR) and CD28 in the presence of CM from mesothelial cells treated with IL‐17A as well as TNF or untreated mesothelial cells (Figure 6D, S11A). As shown in Figure 6E and F, CM from cytokine‐treated cells strongly amplified the abundance of IL‐17A‐producing CD4^+^ cells compared to CM from untreated cells. As a control, CD4^+^ lymphocytes were cultured in medium supplemented with IL‐1β, IL‐6, IL‐23 and TGFβ, which showed a weaker effect (Figure S11B and C). These results demonstrate enhanced Th17 differentiation mediated by the secretome of mesothelial cells reprogrammed by IL‐17A and TNF. Of note, CCL20 is also elevated in this reprogrammed mesothelial secretome (Figure S10). CCL20 is the ligand for the homing receptor CCR6 on Th17 cells, mediating Th17 cell recruitment into a CCL20‐rich environment,^21^, ^57^ thereby complementing the differentiation‐promoting effect of IL‐1β and IL‐6. Taken together, these data indicate a positive feedback loop established by the reciprocal crosstalk of Th17 and mesothelial cells.

## 4 DISCUSSION

A decisive step in the metastatic cascade of transcoelomic dissemination by OC cells is breaching the protective mesothelial monolayer covering the peritoneal organs. As schematically summarized in the Graphical Abstract, the present study uncovered a previously unknown mechanism involving the induction of mesenchymal reprogramming by the synergistic action of IL‐17A and TNF produced by Th17 cells, which renders the mesothelial monolayer susceptible to cancer cell adhesion. We also show that mesothelial cells undergoing MMT secrete Th17‐promoting mediators, thereby establishing a positive feedback loop that may amplify the observed effects. While the role of TNF in mesenchymal reprogramming is well known, an involvement of Th17 cells in cancer‐associated MMT has not been reported to date. We therefore subsequently discuss our findings with a focus on IL‐17A, its synergism with TNF and the promotion of tumor cell adhesion to the mesothelium.

### 4.1 Diverse functions of IL‐17A in OC

IL‐17A is produced by a broad range cell types, including CD4^+^, CD8^+^, γδ T cells and various innate immune cell populations including MAIT cells.^21^, ^69^ In OC, the role of IL‐17A producers complex, including both tumor‐promoting and tumor‐suppressive functions, and effecting diverse biological processes. In the context of tumor promotion, several studies have reported an increased abundance of Th17 cells in the OC TME,^30^, ^72^, ^73^ and the abundance of tumor‐infiltrating IL‐17A‐producing producing γδ T cells correlated with larger tumor size and lymph node metastasis.^73^ Furthermore, IL‐17 signaling has been linked to the recruitment of myeloid cells to the OC TME.^19^, ^29^ These observations are in line with our data indicating the production of numerous myeloid‐cell‐targeting cytokines and chemokines by IL‐17A‐treated mesothelial cells, including multiple members of the CCL and CXCL families, interleukins and CSF3, which is synergistically enhanced by TNF. It is likely that these mediators promote the recruitment of immune cells and their pro‐tumorigenic programming in the OC TME. The neutrophil‐promoting CSF3 (G‐CSF) may be of particular interest in this context in view of its strong induction, the reported role of neutrophil extracellular traps in ovarian cancer premetastatic niche formation,^74^ as well as the neutrophil‐mediated suppression of T cell‐mediated immune surveillance.^75^

A key finding of our study is the synergistic induction of MMT by IL‐17A+TNF as a potentially crucial step in peritoneal metastasis, which is supported by RNA‐Seq analysis of cultured patient‐derived mesothelial cells, scRNA profiling of cells obtained from metastasized omentum of OC patients as well as functional assays in vitro and in an ex vivo mouse model. The involvement of IL‐17A in MMT has not been reported to date. Consistent with our data, however, the promotion of the related EMT and EMT‐associated biological processes by IL‐17A have been described for OC as well as other tumor entities. These include, for example, the promotion of (i) EMT of lung cancer cells,^24^ (ii) the induction of stem‐cell features of pancreatic and ovarian carcinoma cells,^25^, ^26^ and (iii) stromal remodeling in pancreatic carcinoma involving the transition of fibroblasts towards an inflammatory cancer‐associated phenotype.^23^

These observations provide overwhelming evidence for a tumor‐promoting impact of IL‐17A in OC. However, in contrast to this conclusion, a number of studies suggest a tumor‐suppressive role for IL‐17A in OC.^27^, ^31^, ^76^, ^77^, ^78^ As our survival analyses indicate an association of both *IL17A* mRNA expression in tumor tissue and IL‐17RC levels in ascites with a short survival, the pro‐tumorigenic functions of IL‐17A appear to predominate in determining the clinical outcome.

### 4.2 Induction of MMT by IL‐17 and TNF

Tumor cell adhesion to the mesothelium and their migration through the mesothelial monolayer into the underlying tissue is a pivotal step in OC metastasis. Several mechanisms affecting the differentiation and function of mesothelial cells in the TME have been proposed in this context. Besides their clearance by tumor‐cell‐mediated mechanical force^10^ or induction of apoptosis,^12^ their reprogramming by MMT appears to be of particular relevance.^11^, ^14^, ^15^, ^79^, ^80^, ^81^ MMT is a multi‐stage process involving the loss of intercellular junctions, dysregulation of cell adhesion molecules, enhancement of migratory properties, synthesis of extracellular matrix (ECM) components, secretion of pro‐inflammatory proteins and upregulation of EMT‐related transcription factors.^61^, ^81^, ^82^ These hallmarks of MMT were all replicated in mesothelial cells exposed to IL‐17A+TNF, displaying a loss of tight junctions, induction of genes involved in cell adhesion and ECM remodeling (*CDH2, MMP2, FN1*, collagens and laminins), upregulation of EMT‐driving transcription factor genes (*SNAI1*, *SNAI2* and *ZEB1), as well as* enhanced expression of proinflammatory cytokines. Conversely, the epithelial markers *KRT19* and *CDH1* were down‐regulated, consistent with mesenchymal skewing.

This phenotypic shift is also evident from a loss of the highly specific mesothelial marker *ITLN1* in a subset of mesothelial cells in the omentum of OC patients, as shown by scRNA‐Seq, supporting the view of IL‐17+TNF‐induced MMT as a specific program distinct from EMT. Importantly, the observed reprogramming observed with cultured mesothelial cells exposed to IL‐17A+TNF was mirrored in a model of explanted mouse omentum by a strong upregulation of the mesenchymal marker VCAM‐1. Given the complexity of MMT, any population of mesothelial cells undergoing mesenchymal reprogramming is likely to represent a continuum of cells in various stages of differentiation and a spectrum of functional properties. This is reflected in our scRNA‐Seq data showing coexpression of mesothelial and mesenchymal marker genes in the majority of patient‐derived mesothelial cells, and is also supported by the observed interpatient heterogeneity.

### 4.3 Promotion of OC cell adhesion to mesothelial cells by IL‐17 and TNF

It has been proposed that the adhesion of OC cells to the mesothelium may be mediated by cell‐cell contacts involving specific receptor‐ligand interactions.^82^ These include the binding of CD44 on OC cells to the glycosaminoglycan hyaluronan on mesothelial cells,^83^, ^84^, ^85^ even though hyaluronic acid secreted by mesothelial cells has also been suggested to provide a barrier to tumor cell adhesion.^86^ Hyaluronan is synthesized by specific synthases, one of which is induced by IL‐17A+TNF at the level of transcription (*HAS2*; Table S4). It is therefore possible that the promotion of tumor cell adhesion of mesothelial cells by IL‐17A+TNF is partly mediated by an enhanced interaction of CD44 with hyaluronan. Other proteins on mesothelial cells proposed to interact with surface proteins on OC cells are VCAM‐1, L1CAM, mesothelin (*MSLN*) and P‐selectin (*SELP*).^82^ None of the corresponding genes was, however, induced by IL‐17A+TNF, suggesting a minor role, if any, in the enhancement of adhesion.

Adhesion of tumor cells may also occur through binding to the collagen‐rich ECM at discontinuous regions in the mesothelial monolayer, either at naturally occurring milky spots^87^, ^88^ or induced by tumor and tumor‐associated host cells.^89^ Our data show that IL‐17A+TNF induced an elongated fibroblast‐like phenotype concomitantly with small gaps in the monolayer, which is consistent with the previously described retraction of mesothelial cells triggered by inflammatory cytokines.^9^, ^15^ It is therefore conceivable that IL‐17A+TNF promote the adhesion of OC cells by inducing the retraction of mesothelial cells, thereby disrupting the mesothelial monolayer to expose the underlying ECM components, which are also secreted by IL‐17A+TNF‐stimulated mesothelial cells (see above). This breaching of the mesothelial monolayer may be initiated and/or facilitated by an increased adhesion of tumor cells to the mesothelium by receptor‐ligand interactions as discussed above.

### 4.4 A positive feedback loop promoting Th17 differentiation by reciprocal crosstalk of Th17 and mesothelial cells

Our affinity proteomic analysis identified several Th17‐promoting mediators in the secretome of IL‐17A+TNF‐stimulated mesothelial cells, notably IL‐6 and IL‐1β as differentiation‐inducing cytokines and CCL20 as a Th17‐attracting chemokine.^21^, ^57^, ^69^, ^71^ These findings hint at a positive autoregulatory loop: mesothelial‐cell‐derived mediators promote Th17 differentiation and homing to peritoneal tumor sites, where these Th17 cells in turn trigger mesothelial cell reprogramming to reinitiate the loop. This model is supported by our observation that CM from IL‐17A+TNF‐treated mesothelial cells strongly enhanced the differentiation of Th17 cells from CD4^+^ T cells from peripheral blood relative to CM from untreated cells. The potential clinical relevance of this interplay is suggested by the abundance of IL‐6, IL‐1β and CCL20 in OC ascites relative to plasma.^56^

### 4.5 Conclusions and limitations of the study

Our data suggest a reciprocal crosstalk between IL‐17A and TNF‐producing Th17 cells and mesothelial cells, resulting in mesenchymal reprogramming of the latter, which in turn drives Th17 cell differentiation and recruitment. This reprogramming of mesothelial cells promotes the adhesion of OC cells to the mesothelial monolayer, suggesting an involvement of IL‐17A and TNF secreted by Th17 cells, as well as the Th17‐mesothelial‐cell amplification loop in omental metastases formation. The abundance of cytokines involved in these interactions in OC ascites (IL‐17A, TNF, IL‐6, IL‐1β, CCL20, TGFβ), the inverse association of several of these mediators with OC survival (IL‐17A, IL‐17RC, IL‐6, TGFβ) and the increased occurrence of Th17 cells in the TME of OC patients indicate that the model suggested by the present study may be clinically relevant. Disruption of this reciprocal crosstalk, for example by neutralizing antibodies, may thus represent a promising strategy to interfere with metastatic spreading in OC patients. A variety of those antibodies are already in clinical use for other diseases, such as infliximab^19^ against TNF, secukinumab^90^ against IL‐17A and olokizumab^91^ against IL‐6, suggesting that their further clinical evaluation for the treatment of OC is feasible.

Although our immunohistochemical analyses of clinical specimens, scRNA‐seq data and observations with explanted mouse omentum are consistent with a potential role of IL‐17A+TNF‐induced MMT in metastasis formation, functional in vivo validation is required to test this model. A considerable obstacle in this context is the complexity of OC mouse models that closely mirror the human disease both genetically and biologically. Even though such models have been described,^92^, ^93^, ^94^ they require complex breeding due to multiple genetic alterations. The introduction of a further gene disruption targeting, e.g., IL17A in Th17 cells or its receptor in mesothelial cells will add another level of complexity. Nevertheless, albeit beyond the scope of the present study, these models may prove useful for future investigations. Another open question of the present study concerns the mechanistic significance of MMT for OC cell invasion through the mesothelium. As summarized in the Background section, several mechanisms mediating disruption of the mesothelial layer have been described, including mechanical force exerted by tumor cells^10^ and the induction of mesothelial cell apoptosis by tumor or tumor‐associated natural killer cells.^12^, ^13^ It remains to be investigated to what extent these different mechanisms contribute to metastasis formation in OC patients. Comprehensive immunohistochemical analyses of clinical samples could provide clues in this direction.

## AUTHOR CONTRIBUTIONS

FN performed the experiments in Figures 1A ‐D, 2, 3, 5 and the corresponding Supplementary Figures; SL mouse and Th17 differentiation experiments; CK and MMG immunohistochemical studies. FN, VS AMS and TW cooperated in adhesion experiments; HR and SL performed FACS analyses, KR supervised microscopic studies; AB provided reagents and analyzed data; VMB and JG carried out affinity proteomics and processed the data; KP and HG prepared samples for scRNA‐Seq; JTS, AN and TS performed RNA‐Seq; FF and RM carried out bioinformatic analyses; MH, RM and SR conceived the study and oversaw the project; MH, RM, SL and FN wrote the paper. All authors read and approved the final manuscript.

## CONFLICT OF INTEREST STATEMENT

The authors declare no conflict of interest.

## ETHICS APPROVAL AND CONSENT TO PARTICIPATE

All experiments were carried out with informed consent by the patients and approval by the ethics committee of Marburg University (205/10).

## CONSENT FOR PUBLICATION

All patients have agreed in writing to the publication of pseudonymized data derived from clinical materials.

## Supporting information

Supporting Information

Supporting Information

## Data Availability

All data generated or analyzed in this study are included in the supplementary files or have been deposited as indicated in the text.
