# Reciprocal crosstalk between Th17 and mesothelial cells promotes metastasis‐associated adhesion of ovarian cancer cells

**DOI:** 10.1002/ctm2.1729

**Published:** 2024-05-31

https://onlinelibrary.wiley.com/doi/10.1002/ctm2.1604

Erratum for Research Article

By Felix Neuhaus et al.

During the revision process, Julia Teply‐Szymanski was inadvertently omitted as a coauthor. We apologise for this error.

Original version of author list:

Felix Neuhaus^1,2^ | Sonja Lieber^1^ | Veronika Shinkevich^3^ | Anna Mary Steitz^2^ | Hartmann Raifer^1,4^ | Kathrin Roth^5^ | Florian Finkernagel^6^ | Thomas Worzfeld^3,7^ | Andreas Burchert^8^ | Corinna Keber^9^ | Andrea Nist^10^ | Thorsten Stiewe^10^ | Silke Reinartz^2^ | Vanessa M. Beutgen^11^ | Johannes Graumann^11^ | Kim Pauck^12^ | Holger Garn^12^ | Matthias Gaida^13,14,15^ | Rolf Müller^2^ | Magdalena Huber^1^

Corrected version of author list:

Felix Neuhaus^1,2^ | Sonja Lieber^1^ | Veronika Shinkevich^3^ | Anna Mary Steitz^2^ | Hartmann Raifer^1,4^ | Kathrin Roth^5^ | Florian Finkernagel^6^ | Thomas Worzfeld^3,7^ | Andreas Burchert^8^ | Corinna Keber^9^ | Julia Teply‐Szymanski^10^ | Andrea Nist^10^ | Thorsten Stiewe^10^ | Silke Reinartz^2^ | Vanessa M. Beutgen^11^ | Johannes Graumann^11^ | Kim Pauck^12^ | Holger Garn^12^ | Matthias Gaida^13,14,15^ | Rolf Müller^2^ | Magdalena Huber^1^

