# Supplementary material for: Reciprocal crosstalk between Th17 and mesothelial cells promotes metastasis‐associated adhesion of ovarian cancer cells
Source: Clin Transl Med. 2024 Apr 2;14(4):e1604. doi: 10.1002/ctm2.1604 (PMC10988119; doi:10.1002/ctm2.1604)
Supplement: Supplementary file 1 — Supporting Information [file CTM2-14-e1604-s002.docx]

Reciprocal crosstalk between Th17 and mesothelial cells promotes metastasis-associated adhesion of ovarian cancer cells

Felix Neuhaus[1,2], Sonja Lieber[1], Veronika Shinkevich[3], Anna Mary Steitz[2], Hartmann Raifer[1,4], Kathrin Roth[5], Florian Finkernagel[6], Thomas Worzfeld[3,7], Andreas Burchert[8], Corinna Keber[9], Andrea Nist[12], Thorsten Stiewe[12], Silke Reinartz[2], Vanessa M. Beutgen[13], Johannes Graumann[13], Kim Pauck[14], Holger Garn[14], Matthias Gaida[9,10,11], Rolf Müller[2*], Magdalena Huber[1*]

[1] Institute of Systems Immunology, Center for Tumor Biology and Immunology (ZTI), Philipps University, Marburg, Germany

[2] Department of Translational Oncology, Center for Tumor Biology and Immunology (ZTI), Philipps University, Marburg, Germany

[3] Institute of Pharmacology, Philipps University, Marburg, Germany

[4] FACS Core Facility, Center for Tumor Biology and Immunology (ZTI), Philipps University, Marburg, Germany

[5] Cell Imaging Core Facility, ZTI, Philipps University, Marburg, Germany

[6] Bioinformatics Core Facility, ZTI, Philipps University, Marburg, Germany

[7] Department of Pharmacology, Max Planck Institute for Heart and Lung Research, Bad Nauheim, Germany

[8] Department of Hematology, Oncology and Immunology, University Hospital Giessen and Marburg, 35043 Marburg, Germany

[9] Comprehensive Biomaterial Bank Marburg (CBBMR) and Institute of Pathology, Philipps University, Marburg, Germany

[10] Institute of Pathology and Research Center for Immunotherapy, University Medical Center Mainz, Mainz, Germany

[11] Joint Unit Immunopathology, Institute of Pathology, University Medical Center, and TRON Translational Oncology Center, Johannes Gutenberg University, Mainz, Germany

[12] Genomics Core Facility, Institute of Molecular Oncology, Member of the German Center for Lung Research (DZL), Philipps University, Marburg, Germany

[13] Institute of Translational Proteomics and Translational Proteomics Core Facility, Biochemical Pharmacological Centre, Philipps University, Marburg, Germany

[14] Translational Inflammation Research Division and Core Facility for Single Cell Multiomics, Philipps University, Marburg, Germany

[*]Correspondence: rolf.mueller@uni-marburg.de, hubermag@staff.uni-marburg.de

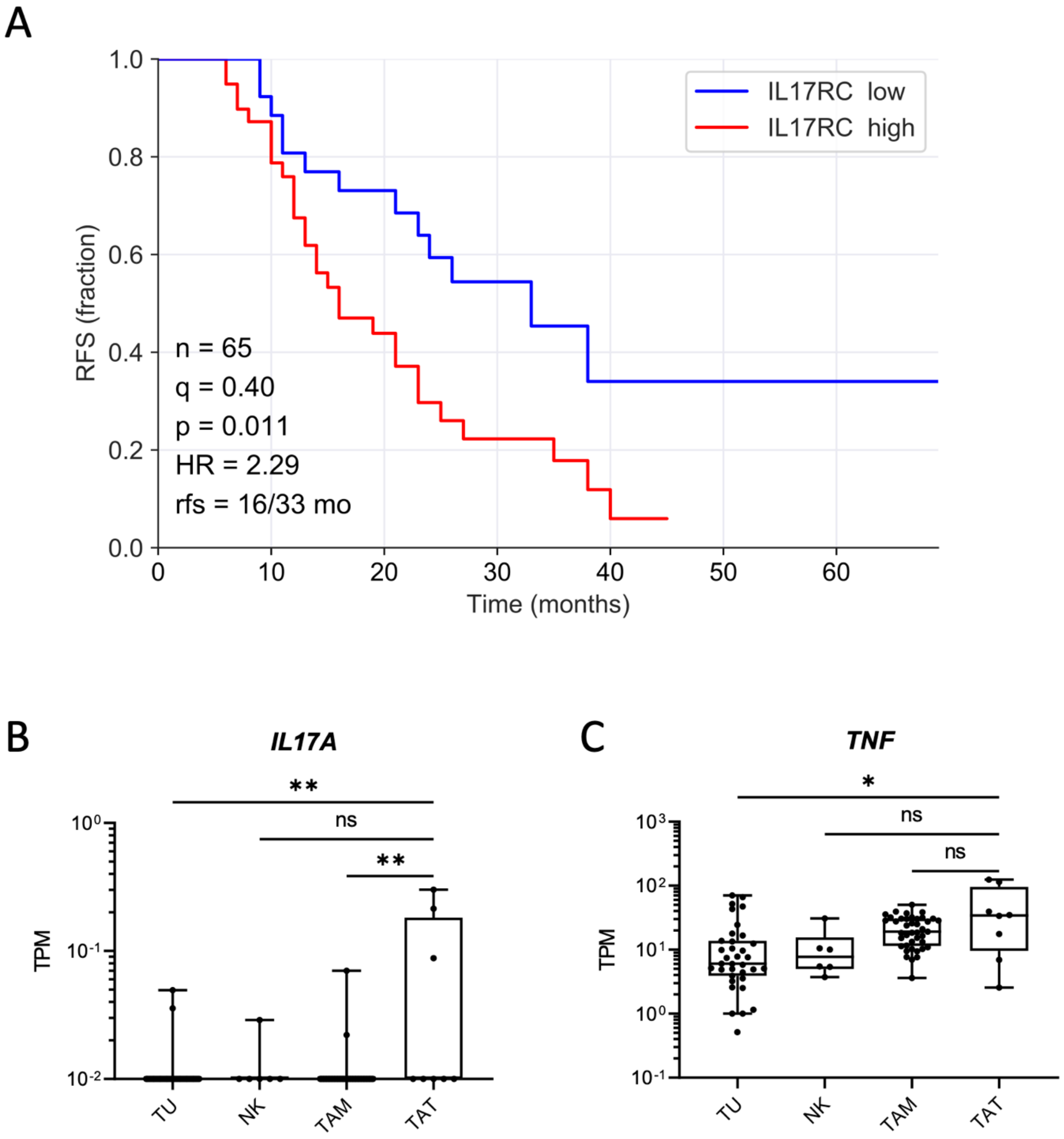

Figure S1. Clinical associations of IL-17 and TNF signaling with OC.

(A) Kaplan Meier plot illustrating an inverse correlation of on IL-17RC protein levels measured by affinity proteomics[51] with the relapse-free survival (RFS) of OC patients (n=65). q: best-fit quantile for splitting low and high expressors; p: longrank p value; HR: hazard ratio; rfs: RFS for high/low IL-17RC expressing patients.

(B, C) Expression levels of *IL17A* and *TNF* genes in cells isolated from OC ascites. TU: Tumor cells, NK: NK cell, TAM: tumor-associated macrophages, TAT: tumor-associated T cells. Boxplots show mean, minimum, maximum and quantiles of biological replicates (n=6-41) and are based on our published RNA-Seq data.[8] Boxplots show the mean, minimum, maximum, and quantiles; biological replicates are plotted. *p<0.05, **p<0.01 were determined by Kruskal-Wallis test followed by Dunn's multiple comparison test. ns: not significant.

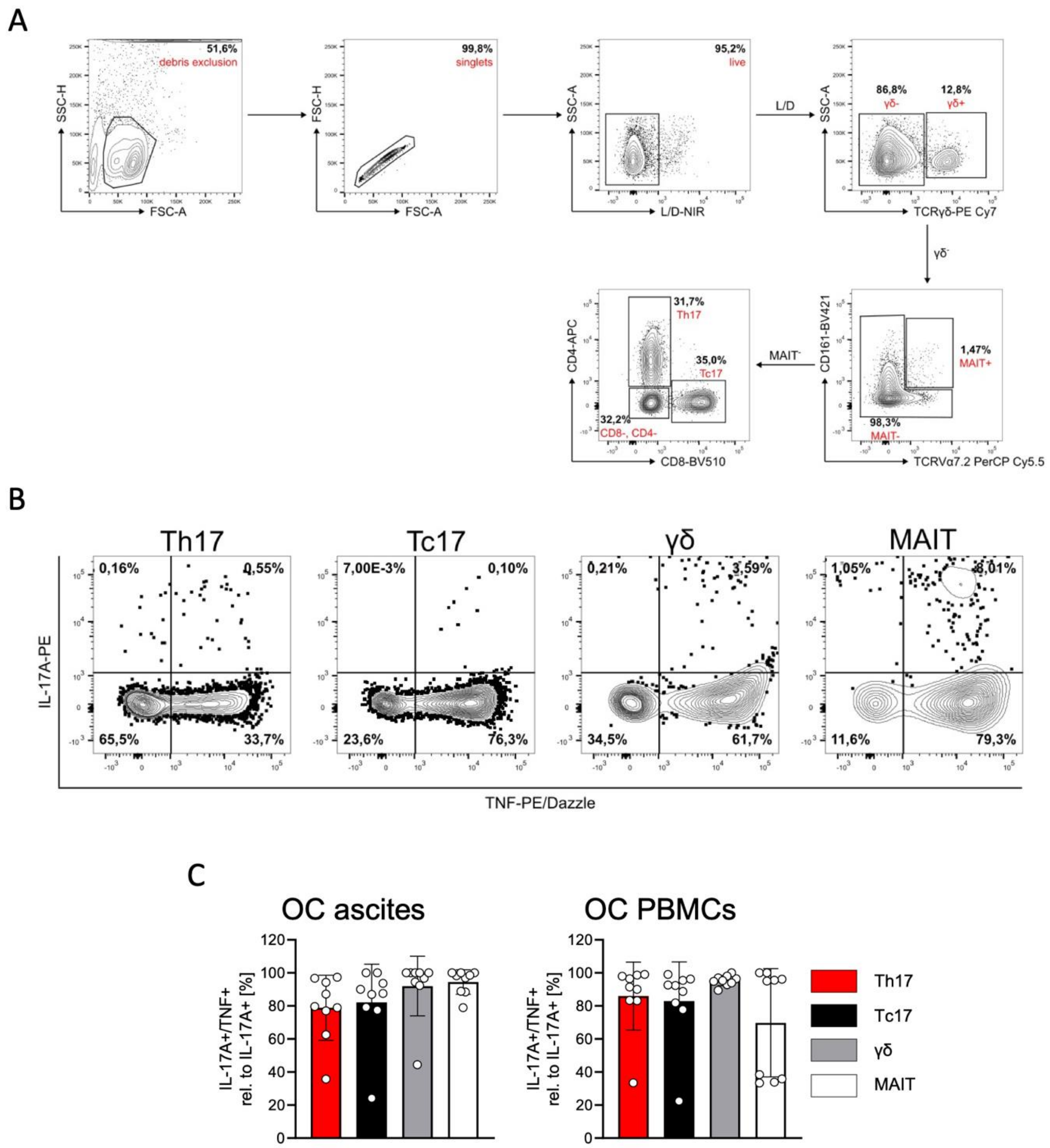

Figure S2. Flow cytometric analysis of CD14-depleted OC PBMCs and ascites cells for specific markers of IL-17A-producing T cells.

(A) Gating strategy for FACS analysis of $CD14^-$ ascites cells after restimulation with α-CD28, ionomycin and PMA in the presence of Brefeldin A for 16 h. The acquired cells were gated for exclusion of debris (SSC-A vs FSC-A), then for single cells (FSC-H vs FSC-A) and finally for living cells (L/D vs SSC-A). γδ T cells were identified by the expression of TCRγδ. $TCR\gamma\delta^-$ were then gated for CD161 and TCRVα7.2 double-positive cells to identify MAIT cells. Finally, MAIT-negative cells were gated for CD8 and CD4 positivity to identify Th17 and Tc17 cells.

(B) FACS analysis for IL-17A and TNF after restimulation of $CD14^-$ ascites cells with α-CD28, ionomycin and PMA in the presence of Brefeldin A for 16 h. Representative plots are shown for Th17 , Tc17, γδ, and MAIT cells.

(C) Percentages of $IL\text{-}17A^+/TNF^+$ double-positive cells relative to $IL\text{-}17A^+$ single-positive cells in $CD14^-$ OC ascites cells and PBMCs (n=9).

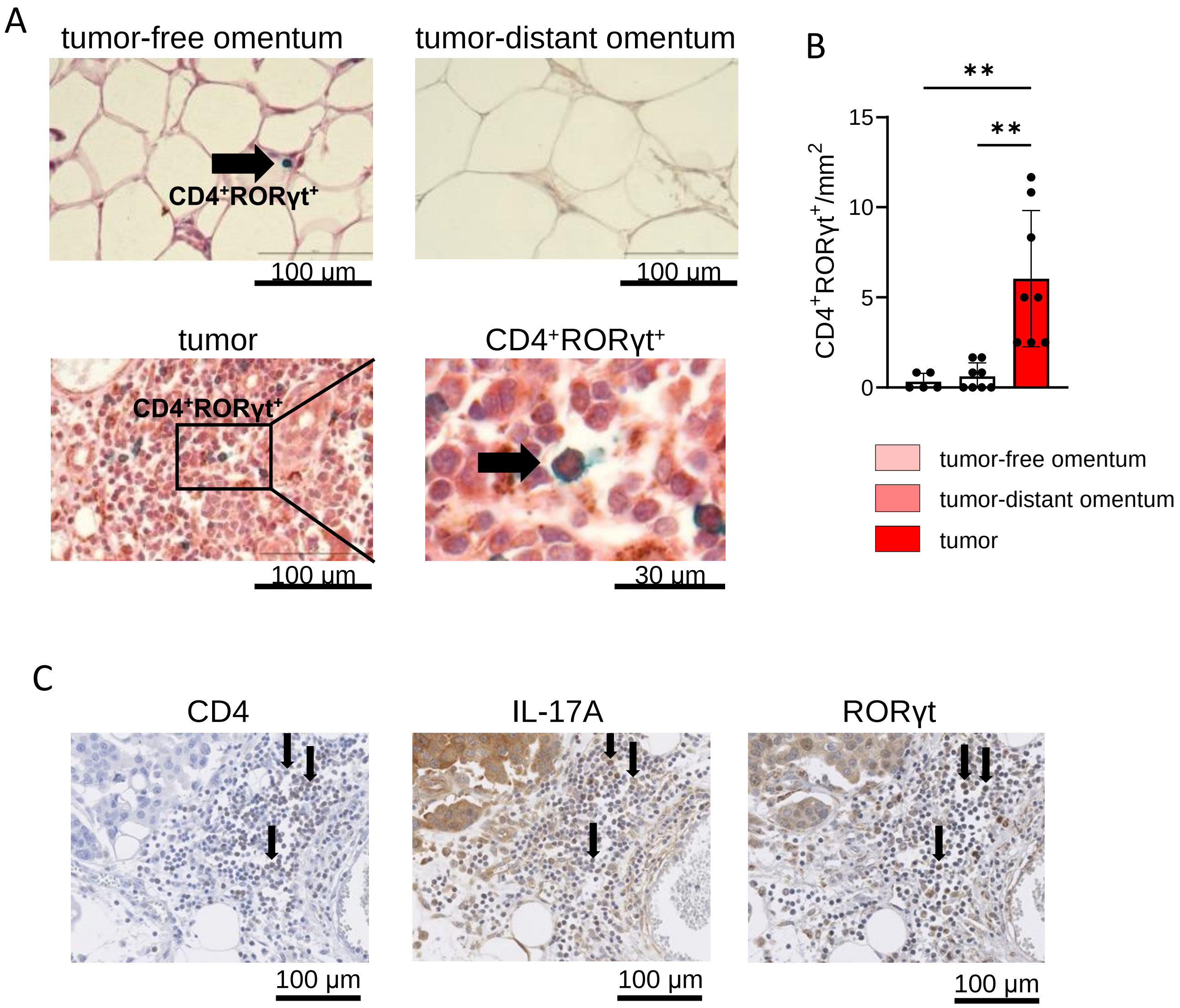

Figure S3. Immunohistochemistry for CD4 and RORγt expression in omentum and micrometastases

(A) Representative pictures showing immunostaining of CD4 (green) and RORγt (brown) in tumor-free omentum (n=5), tumor-distant omentum and tumor tissue in omental micrometastases (n=8). Arrows indicate $CD4^+$/$ROR\gamma t^+$ double-positive cells.

(B) Quantification of $CD4^+$/$ROR\gamma t^+$ double-positive cells in tumor-free omentum (n=5), tumor distant, or tumor tissue in omental micrometastases (n=8). Bar plot indicates the mean±SD of biological replicates. **p<0.01 were determined by Kruskal-Wallis followed by Dunn's multiple comparison test.

(C) Representative pictures showing single staining for CD4, IL-17A and RORγt in omental mcirometastases.

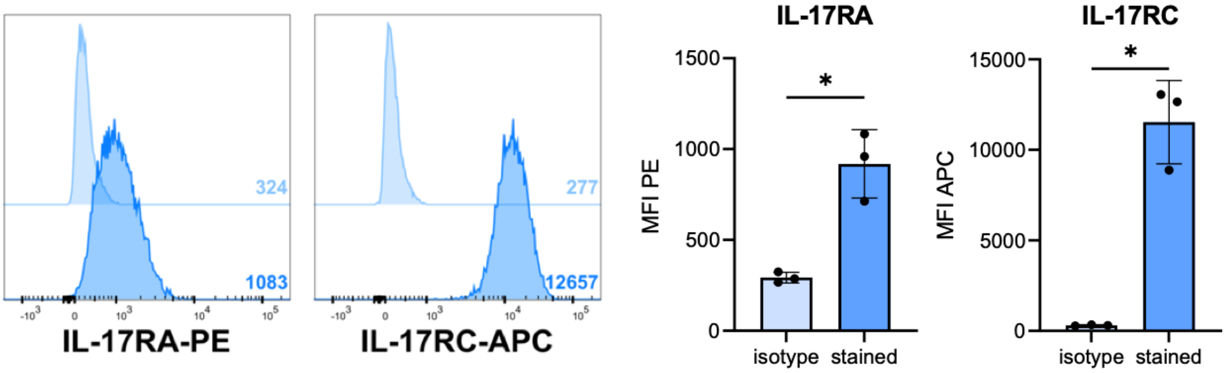

Figure S4. FACS analysis of IL-17RA and IL-17RC expression in omental mesothelial cells.

Left: representative histogram; right: quantification (MFI) of histograms. *$p<0.05$ was determined by two-tailed paired t-test.

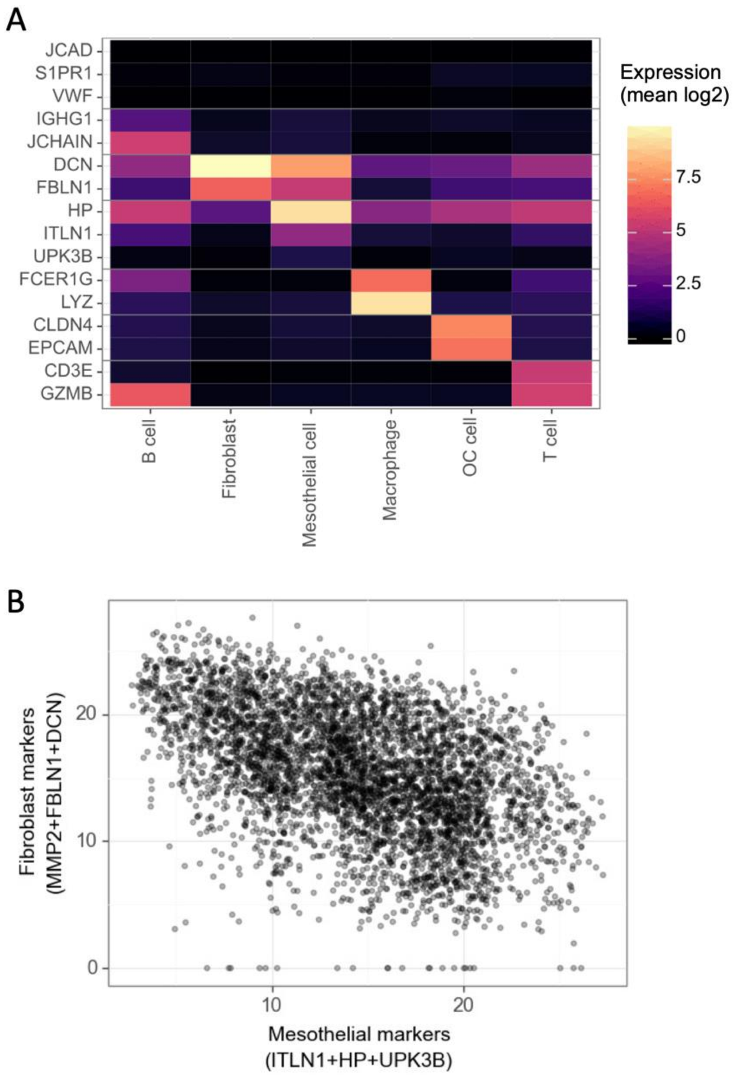

Figure S5. Cell-type-selective marker gene expression in enriched mesothelial cell fractions from the omenta of OC patients (targeted scRNA-Seq).

(A) Heatmap of marker genes expressed in Louvain clusters (scRNA-Seq). Two-dimensional embedding of scRNA-Seq data by t-SNE, clustering via Louvain and annotation to cell types were performed as in Figure 4B.

(B) Scatter plot of mesothelial and fibroblast marker gene expression by mesothelial cells identified in Fig. 4B. X-axis: sum of log2 gene expression of the indicated mesothelial marker genes (also used for bioinformatic identification of mesothelial cells in Fig. 4). Y-axis: sum of log2 gene expression of fibroblast marker genes. Pearson correlation r^2 = -0.47.

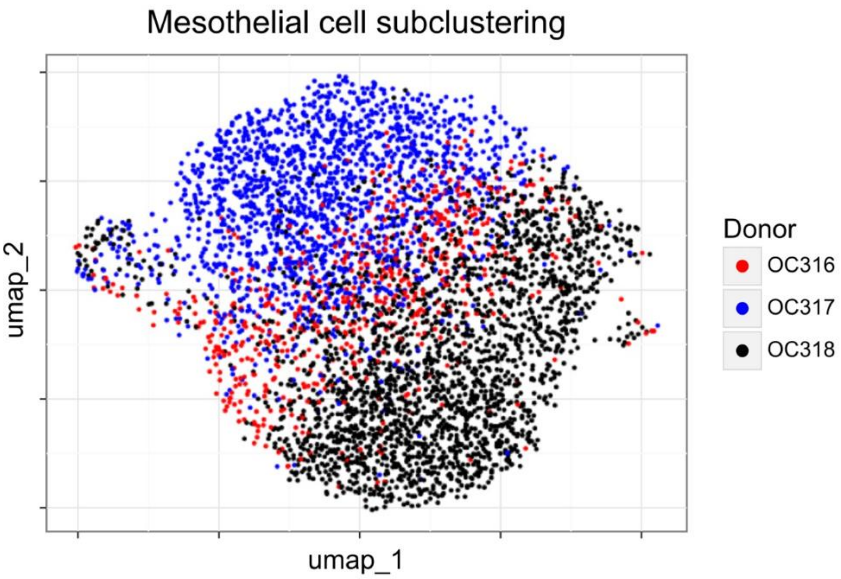

Figure S6. Donor dependence of Louvain clusters of IL-17A/TNF-induced genes (scRNA-Seq). Two-dimensional embedding (UMAP) and Louvain clustering of mesothelial cells was performed as in Figure 4C for individual patients (different colors)

A

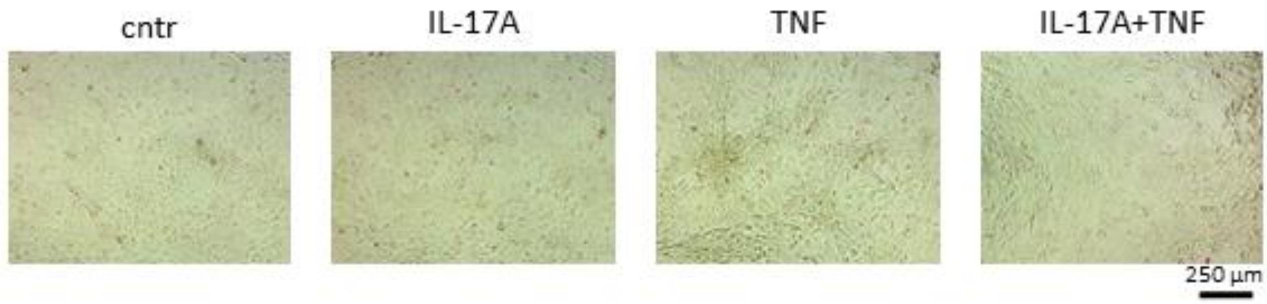

B

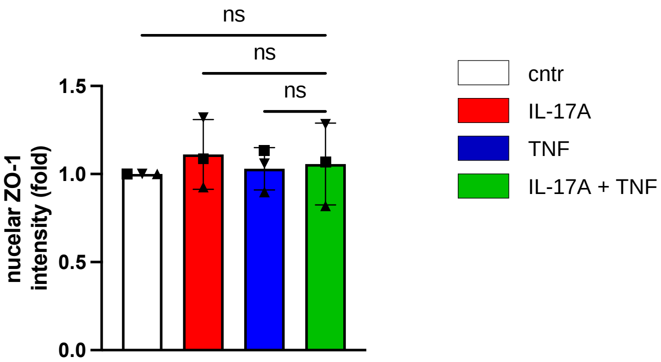

Figure S7. Representative pictures showing mesothelial monolayer integrity before addition of tumor cells.

(A) Cells were treated as indicated and analyzed by bright-field microscopy.

(B) Nuclear ZO-1 intensity was quantified using the ImageJ software. Nuclei were defined by positivity for Hoechst staining.

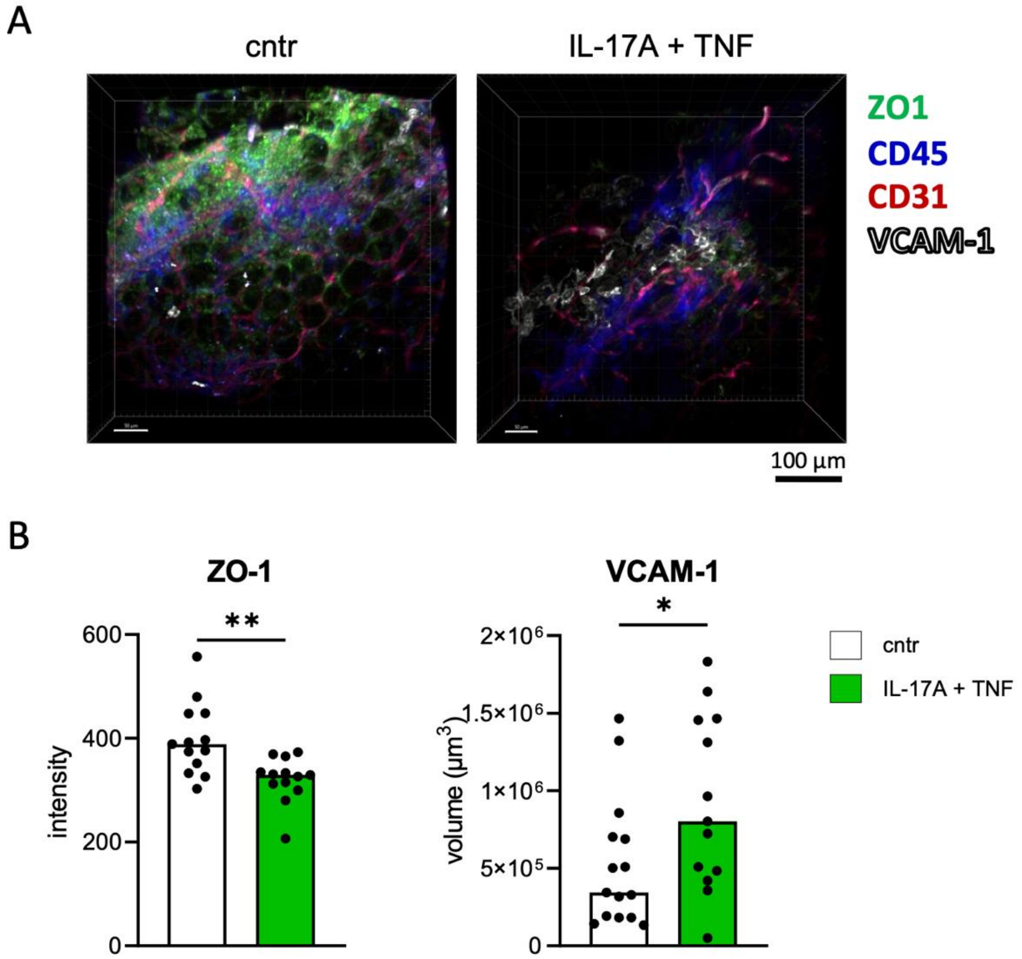

Figure S8. IL-17A/TNF alter the phenotype of mesothelial cells and perturb monolayer integrity in an ex vivo model of mouse omentum.

(A) Whole mount staining of mouse omentum cultured *ex vivo* in medium with or without with rmIL-17A (30ng/ml) plus rmTNF (10ng/ml). Representative pictures taken after 24 h of incubation are shown. Immunostaining was performed for ZO-1 (green), VCAM-1 (white), CD31 (red) and CD45 (blue).

(B) Quantification of ZO-1 intensity and VCAM-1 volume by analysis of random fields for both conditions using Imaris software. Bar plots show the mean±SD; data points (counts per field) of three independent experiments using different omenta are plotted. *$p<0.05$, **$p<0.01$ were determined by two-tailed unpaired t-test.

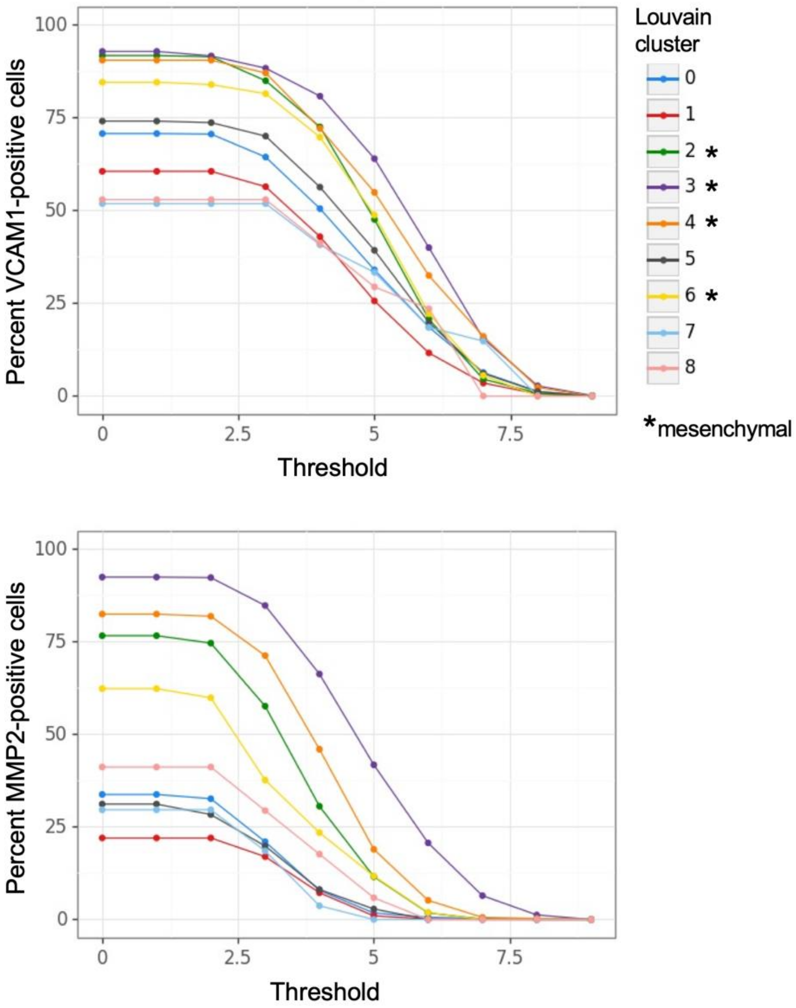

Figure S9. Analysis of scRNAseq data for *VCAM1*- and *MMP2*-postive cells in Louvain clusters identified in Fig. 4. The plots show the percentage of positive cells per cluster that exceed the indicated threshold (log2 reads normalized to 10.000 per cell).

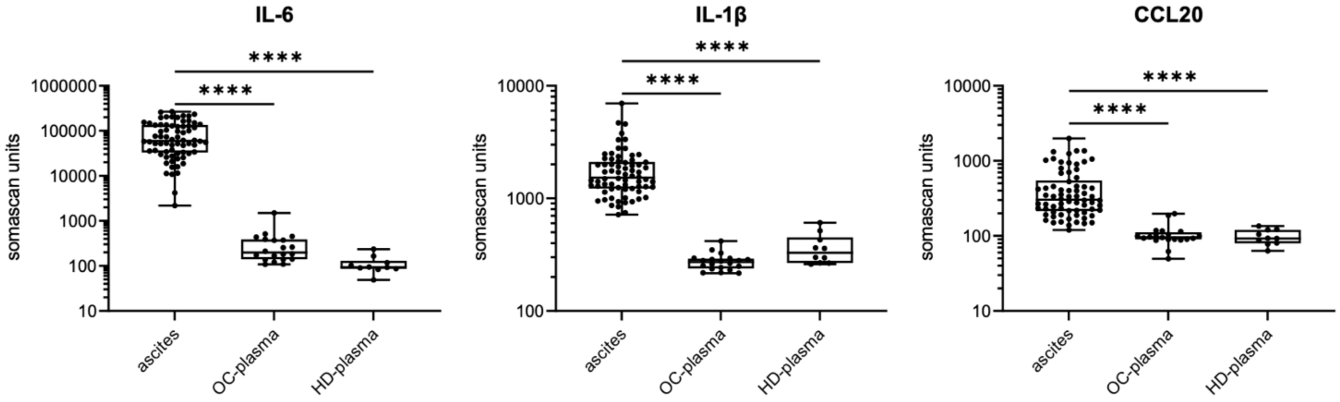

Figure S10. Increased levels of Th17 driving cytokines in ascites.

SOMAscan signals for IL-6, IL-1β and CCL20 in plasma obtained from healthy donors (HD-plasma; n=10), plasma from OC-patients (n=20) and OC ascites: ascites (n=70). Boxplots show mean, minimum, maximum and quantiles of biological replicates and are based on our published affinity proteomics data.[51] ****p<0.0001 determined by Kruskal-Wallis test.

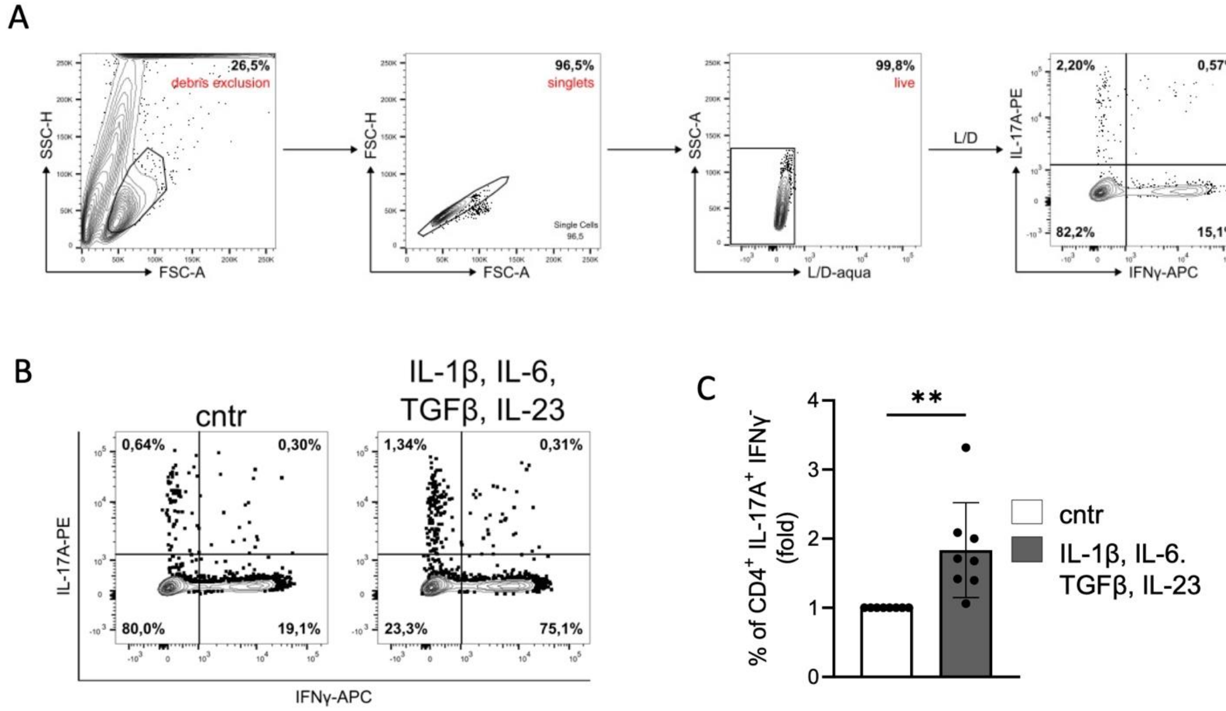

Figure S11. Flow cytometric analysis of $CD4^+$ cells stimulated with differentiation driving cytokines.

(A) Gating strategy for FACS analysis of PMA- and ionomycin-restimulated human $CD4^+$ T cells after differentiation in the presence or absence of Th17-driving cytokines. The acquired cells were gated for exclusion of debris (SSC-A vs FSC-A), then for single cells (FSC-H vs FSC-A) and finally for living cells (L/D vs SSC-A). IL-17A^+IFNγ^- Th17 cells were identified based on the expression of IL-17A or IFNγ.

(B) Exemplary staining for IL-17A and IFNγ after restimulation with PMA and ionomycin in the presence of brefeldin A of $CD4^+$ cells differentiated in the presence of IL-6, IL-1β, and TGFβ compared to $CD4^+$ T cells differentiated in the absence of Th17-driving cytokines.

(C) Fold change of IL-17A^+/IFNγ^- cells after treatment with IL-1β, IL-6, IL-23 and TGFβ compared to untreated cells (n=5). Bar plots show the mean±SD of biological replicates. **$p<0.01$ determined by two-tailed unpaired t-test.
